# Transcriptomic Analysis of the Influence of Methanol Assimilation on the Gene Expression in the Recombinant *Pichia pastoris* Producing Hirudin Variant 3

**DOI:** 10.3390/genes10080606

**Published:** 2019-08-12

**Authors:** Tao Li, Jieying Ma, Zehua Xu, Shuang Wang, Nan Wang, Shulin Shao, Wei Yang, Lin Huang, Yihan Liu

**Affiliations:** 1 Key Laboratory of Industrial Fermentation Microbiology, Ministry of Education, Tianjin Key Laboratory of Industrial Microbiology, The College of Biotechnology, Tianjin University of Science and Technology, Tianjin 300457, China; 2 College of Basic Science, Tianjin Agricultural University, Tianjin 300384, China

**Keywords:** anticoagulant, methanol induction, fermentation, hirudin, *Pichia pastoris*

## Abstract

Hirudin and its variants, as strong inhibitors against thrombin, are present in the saliva of leeches and are recognized as potent anticoagulants. However, their yield is far from the clinical requirement up to now. In this study, the production of hirudin variant 3 (HV3) was successfully realized by cultivating the recombinant *Pichia pastoris* GS115/pPIC9K-*hv3* under the regulation of the promoter of *AOX1* encoding alcohol oxidase (AOX). The antithrombin activity in the fermentation broth reached the maximum value of 5000 ATU/mL. To explore an effective strategy for improving HV3 production in the future, we investigated the influence of methanol assimilation on the general gene expression in this recombinant by transcriptomic study. The results showed that methanol was partially oxidized into CO_2_, and the rest was converted into glycerone-P which subsequently entered into central carbon metabolism, energy metabolism, and amino acid biosynthesis. However, the later metabolic processes were almost all down-regulated. Therefore, we propose that the up-regulated central carbon metabolism, energy, and amino acid metabolism should be beneficial for methanol assimilation, which would accordingly improve the production of HV3.

## 1. Introduction

Leeches (or Hirudinea) which belong to the phylum Annelida are closely related to oligochaetes (e.g., the best-known earthworm), and all have muscular, soft, and segmented bodies [1]. More than 700 species of leech living in marine or freshwater environments have been described [2,3]. Leeches are well-known to us for its blood-feeding animal features due to the secretion of hemotoxic proteins from the salivary glands into the blood streams after biting the host [4,5,6]. Leeches can usually consume over 10 times their weight in blood during feeding periods because of the successful counteraction against host hemostatic processes by those hemotoxic proteins as anticoagulants [7,8]. Given the powerful function in preventing the clotting of blood, the leech anticoagulants have been widely used in clinics as modern medicine, such as for venous congestion treatment and as post-surgical instruments following reconstructive and plastic surgery [9,10,11]. Among those anticoagulants from leech saliva, hirudin and its variants are the most famous and well-studied for its high efficiency in inhibiting thrombin with K_I_ and K_D_ values being in the pico- to femtomolar range [12].

Hirudin, as one of the most potent naturally-occurring anticoagulants, was firstly isolated from the saliva of leech *Hirudo medicinalis* [13]. It can efficiently and specifically bind to thrombin, which converts fibrinogen to fibrin in blood clot formation. Shortly after the release of the amino acid sequence of hirudin, three main subtypes of hirudins were discovered in succession and designated as hirudin variant 1 (HV1), HV2, and HV3, sharing a similar core domain of N-terminal sequence and inhibitory activity against thrombin [14,15,16,17].

Given the huge therapeutic value in clinical application and the limited availability of natural hirudin from leech, many researchers have put effort into improving the productivity and production of hirudin variants by genetic recombination with host-microorganisms, including *Escherichia coli* [18], *Saccharomyces cerevisiae* [19], and *Pichia pastoris* [20]. Among them, HV3 and its mutein have been successfully expressed in *E. coli* [21,22]. However, lack of a mature secretory system is still a critical defect of *E. coli* for heterologous protein secretion although additional signal peptide can direct HV3 to secrete into the medium [23]. Therefore, an eukaryotic host should be more appropriate for the production of HV3 due to the existence of post-translational modifications and a secretory system.

The methylotrophic yeast *P. pastoris* has been widely applied in the heterologous protein expression owing to its many advantages, such as achieving high cell density, post-translational modifications and a secretory system [24]. Three intramolecular disulfide bridges located in the hirudin N-terminal core domain are vital for its antithrombin activity through binding to the active center of thrombin to block its hydrolysis activity [25]. Therefore, the presence of post-translational processing system in *P. pastoris* is much more favored for the expression of hirudin and its variants.

Few reports covering the production of recombinant HV3 using *P. pastoris* have been published up to now. Although *P. pastoris* is a well-developed heterologous protein expression host, some disadvantages about this system are still noteworthy. The clonal variation is likely to be a major bottleneck for the industrial application of this host system as hundreds of clones per phenotype need to be screened to get the highest secretor [26,27]. Previous studies have usually employed two carbon sources, and adopted a two-stage cultivation strategy when culturing *P. pastoris*: Glycerol for biomass production and methanol for foreign protein expression. However, it was difficult for carbon source switching from one to the other at a precise or proper time point [24,28]. Furthermore, a deep and comprehensive insight on the influence of methanol assimilation on the overall metabolism in the recombinant *P. pastoris* is still not yet clear and is necessary to be explored further.

In this study, the recombinant *P. pastoris* GS115/pPIC9K-*hv3* was successfully constructed, and the expression of *hv3* regulated by promoter of *AOX1* encoding alcohol oxidase (AOX) was realized. However, the growth of this recombinant strain slowed down after the initiation of methanol induction. Moreover, our work indicated that the cell growth should be closely related to the production of HV3 in the recombinant *P. pastoris* during the methanol induction phase. Thus, it is quite necessary to investigate the influence of methanol assimilation on the gene expression in general for further developing an effective strategy regarding the enhanced HV3 production by this strain. Hence, our research would provide a new approach for finding out or solving the bottlenecks for enhanced heterologous protein production in *P. pastoris*.

## 2. Materials and Methods

### 2.1. Materials and Reagents

The complete CDS of HV3 (Accession No. KR066905) was obtained from the NCBI website [29]. The gene *hv3* was synthesized by referring to this reported sequence and the codon usage bias in *P. pastoris*, and this optimized sequence was deposited in GenBank (Accession No. MK341489). *E. coli* DH5α and *P. pastoris* GS115 were applied in the plasmid propagation, gene cloning, and the construction of recombinant for *hv3* expression. Empty plasmid pPIC9K (Invitrogen, San Diego, CA, USA), which contains four restriction enzyme sites (*Eco*RI, *Sna*BI, *Avr*II, and *Not*I), was used for the plasmid engineering. *E. coli* DH5α/pUC57-*hv3* obtaining the gene of *hv3* was purchased from the Beijing Genomics Institute (BGI, Beijing, China). The analytical reagents used in the culture and fermentation experiments were purchased from local suppliers.

### 2.2. Construction of Recombinant P. pastoris

The plasmid pUC57-*hv3* was propagated by incubating *E. coli* DH5α/pUC57-*hv3* at 37 °C in LB medium supplemented with ampicillin at a final concentration of 100 μg/mL overnight. pUC57-*hv3* extracted with a plasmid extraction kit (OMEGA; Norcross, GA, USA) was subsequently digested with *Eco*RI and *Not*I, and the obtained fragment of *hv3* was inserted into plasmid pPIC9K to generate pPIC9K-*hv3*. Afterwards, pPIC9K-*hv3* was transformed into *E. coli* DH5α for propagation. The pPIC9K-*hv3* was linearized by *Sac*I and transformed into 100 μL of *P. pastoris* GS115 competent cells by electroporation (1500 V, 25 mF, 200 Ω) using a Bio-Rad Gene Pulser XcellTM (Hercules, CA, USA) [30]. One milliliter of ice-cold sorbitol (1 M) was then mixed with the cells, and 400 μL of the suspension was cultured in minimal dextrose (MD) medium (Invitrogen, San Diego, CA, USA) at 30 °C for 48 h. The transformants were further screened from YPD agar plates (10 g/L yeast extract, 20 g/L peptone, and 20 g/L glucose) prepared with a G418 gradient (1.0, 2.0, 3.0, 4.0, and 5.0 mg/mL). The recombinant colonies containing multiple copies of *hv3* were confirmed by PCR using the primers F: 5’-GACTGGTTCCAATTGACAAGC-3’ and R: 5’-GCAAATGGCATTCTGACATCC-3’. *P. pastoris* GS115 transformants harboring pPIC9K-*hv3* were designated as *P. pastoris* GS115/pPIC9K-*hv3*. *P. pastoris* cell transformed with the empty pPIC9K plasmid was applied as the control and named as *P. pastoris* GS115/pPIC9K.

### 2.3. Fermentation of P. pastoris GS115/pPIC9K-hv3

We performed the cultivation of *P. pastoris* following a reported method and made some minor modifications [31]. Briefly, *P. pastoris* GS115/pPIC9K-*hv3* and *P. pastoris* GS115/pPIC9K were precultured in YPD medium at 30 °C for 24 h, respectively. Afterwards, 2.5 mL of the above culture was pipetted into 50 mL of BMGY medium in a 250 mL Erlenmeyer flask and cultivated at 30 °C for 24 h with shaking at 250 rpm. The cells harvested by centrifugation at 4000× *g* for 5 min were resuspended in BMMY medium to achieve an OD_600_ of 1.0, and were continuously cultured at 30 °C for 5 days to express HV3, with a supplementation of methanol to a final concentration of 1% (*v*/*v*) every 24 h. Then the broth of *P. pastoris* GS115/pPIC9K-*hv3* and *P. pastoris* GS115/pPIC9K were collected for testing the antithrombin activity.

The fermentation of *P. pastoris* GS115/pPIC9K-*hv3* was conducted in a 5 L fermenter (Baoxing, Shanghai, China). The initial fermentation procedure was as follows: 300 mL of *P. pastoris* GS115/pPIC9K-*hv3* culture grown in BMGY medium for 24 h was inoculated into the fermentation tank containing 2.7 L of minimal salts medium: H_3_PO_4_ (85%), 13 mL/L; CaSO_4_2H_2_O, 0.93 g/L; K_2_SO_4_, 18.2 g/L; MgSO_4_, 7.27 g/L; KOH, 10.6 g/L; sodium citrate 2H_2_O, 1.47 g/L; and 2 mL/L PTM1 trace metals solution [32]. Glycerol was used as the sole carbon source and its final concentration was 4% (*w*/*v*). In the initial phase, the fermentation parameters were set as follows: Temperature 30 °C, 30% of dissolved oxygen maintained by automatically controlling the agitation speed, airflow at 4 slpm, pH at 5 kept by automatically adjusting with ammonium hydroxide. The yeast cells were grown in this fermenter for about 18 h until the exhaustion of glycerol, observing a dramatic increase of dissolved oxygen (DO) and slowdown of agitation speed. Afterwards, 250 mL of 50% glycerol was gradually fed into the fermenter with a speed of 50 mL/h, and the pH was maintained at 3. The methanol induction phase was initiated when a sharp increase of DO was observed, a sign of glycerol depletion. And the methanol induction conditions were as follows: The methanol-fed rate was increased from 1 to 6 mL/h in the first 6 h. Afterwards it was set as: 6–16 h, 0.09 mL/min; 16–40 h, 0.18 mL/min; 40–90 h, 0.26 mL/min; 90–136 h, 0.18 mL/min. 270 mL of minimal salts medium was supplemented at 60 and 96 h, respectively.

### 2.4. Antithrombin Activity Analysis

The antithrombin activity of HV3 in the broth of *P. pastoris* GS115/pPIC9K-*hv3* was analyzed following a previous method [33,34]. Briefly, the activity of HV3 was quantitatively measured by titrating a solution of thrombin and expressed as antithrombin units (ATU). One ATU of test sample is defined as the activity to neutralize one NIH unit of thrombin (Sigma, Darmstadt, Germany). The activity analysis was performed as follows: 0.2 mL of 0.05% bovine fibrinogen solution prepared with 50 mM Tris–HCl buffer (pH 7.4) was thoroughly mixed with 0.01–0.1 mL of sample, and then 5 μL of 100 NIH/mL thrombin solution (0.5 NIH unit) was added progressively and mixed gently. The above mixture was incubated at 37 °C for 1 min, and the end point of the titration was considered to be reached when a fibrin clot formed within 1 min. Otherwise, another 5 μL of thrombin solution was added continuously until a fibrin clot was observed. 

### 2.5. cDNA Library Construction and Illumina Sequencing

Three biological replicates were set for each sample. Total RNA was isolated and purified using the RNeasy Mini Kit (QIAGEN, Alameda, CA, USA) and the RNA integrity of each sample was assessed using the RNA Nano 6000 Assay Kit of the Bioanalyzer 2100 system (Agilent Technologies, Santa Clara, CA, USA). And a total of 3 μg RNA per sample was used for the cDNA library preparation. Sequencing libraries were generated using NEBNext^®^ Ultra™ RNA Library Prep Kit for Illumina^®^ (NEB, Ipswich, MA, USA) following the manufacturer’s recommendations, and index codes were added to attribute sequences to each sample. The cDNA fragments of preferentially 150–200 bp in length were purified with the AMPure XP system (Beckman Coulter, Beverly, MA, USA). Lastly, the PCR products were purified (AMPure XP system) and library quality was assessed on the Agilent Bioanalyzer 2100 system (Agilent).

The clustering of the index-coded samples was performed on a cBot Cluster Generation System using the TruSeq PE Cluster Kit v3-cBot-HS (Illumina) following the manufacturer’s instructions. After cluster generation, the library preparations were sequenced on an Illumina Hiseq platform in Novogene (Beijing, China) and 125 bp/150 bp paired-end reads were generated.

### 2.6. De Novo Assembly and Functional Annotation Analysis of Illumina Sequencing

Raw reads were firstly processed through in-house perl scripts. The transcriptome raw data were deposited in SRA database (Accession No. PRJNA509983). Clean reads obtained by removing reads containing adapter, ploy-N, and low-quality reads from raw reads were used for de novo assembly. Reference genome and gene annotation were downloaded directly from the database (NCBI, *Komagataella phaffii* GS115, assembly ASM2700v1). Index of the reference genome was built using Bowtie v2.2.3 and paired-end clean reads were aligned to the reference genome using TopHat v2.0.12. HTSeq v0.6.1 was used to count the reads numbers mapped to each gene, and the expected number of fragments per kilobase of transcript sequence per millions base pairs sequenced (FPKM) of each gene was calculated based on the length of the gene and the count of reads mapped to this gene [35]. Prior to differential gene expression analysis, the read counts for the sequenced library were adjusted by the edgeR program package through one scaling normalized factor [36]. Differential expression analyses of comparison groups (three biological replicates per condition) were performed using the DEGSeq R package v. 1.18.0 [37,38]. The *p*-value was adjusted using Benjamini and Hochberg’s approach for controlling the false discovery rate [39]. An adjusted *p*-value (*p*_adj_) of 0.01 and log_2_ (fold change) of 1 were set as the threshold for screening the significantly expressed genes by DESeq. Gene Ontology (GO) enrichment analysis of differentially expressed genes (DEGs) was implemented by the GOseq R package, in which gene length bias was corrected [40].

## 3. Results

### 3.1. HV3 Fermentation by P. pastoris GS115/pPIC9K-hv3 in 5L Fermenter

**Figure 1:**
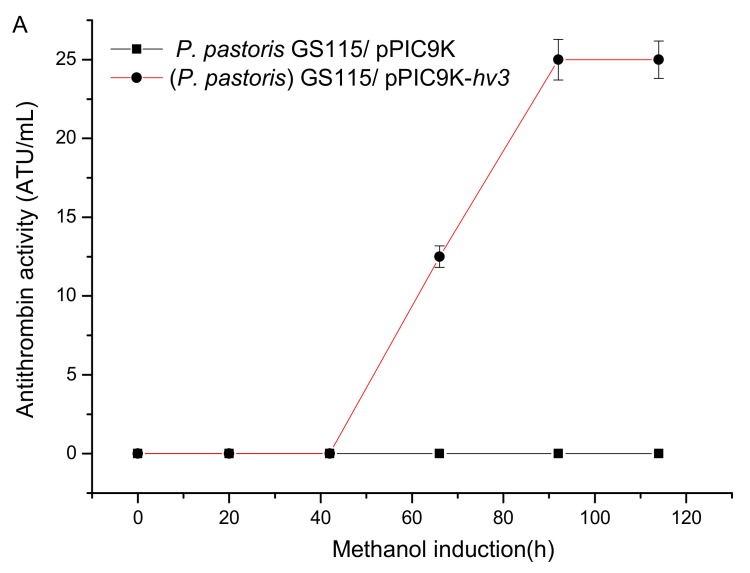

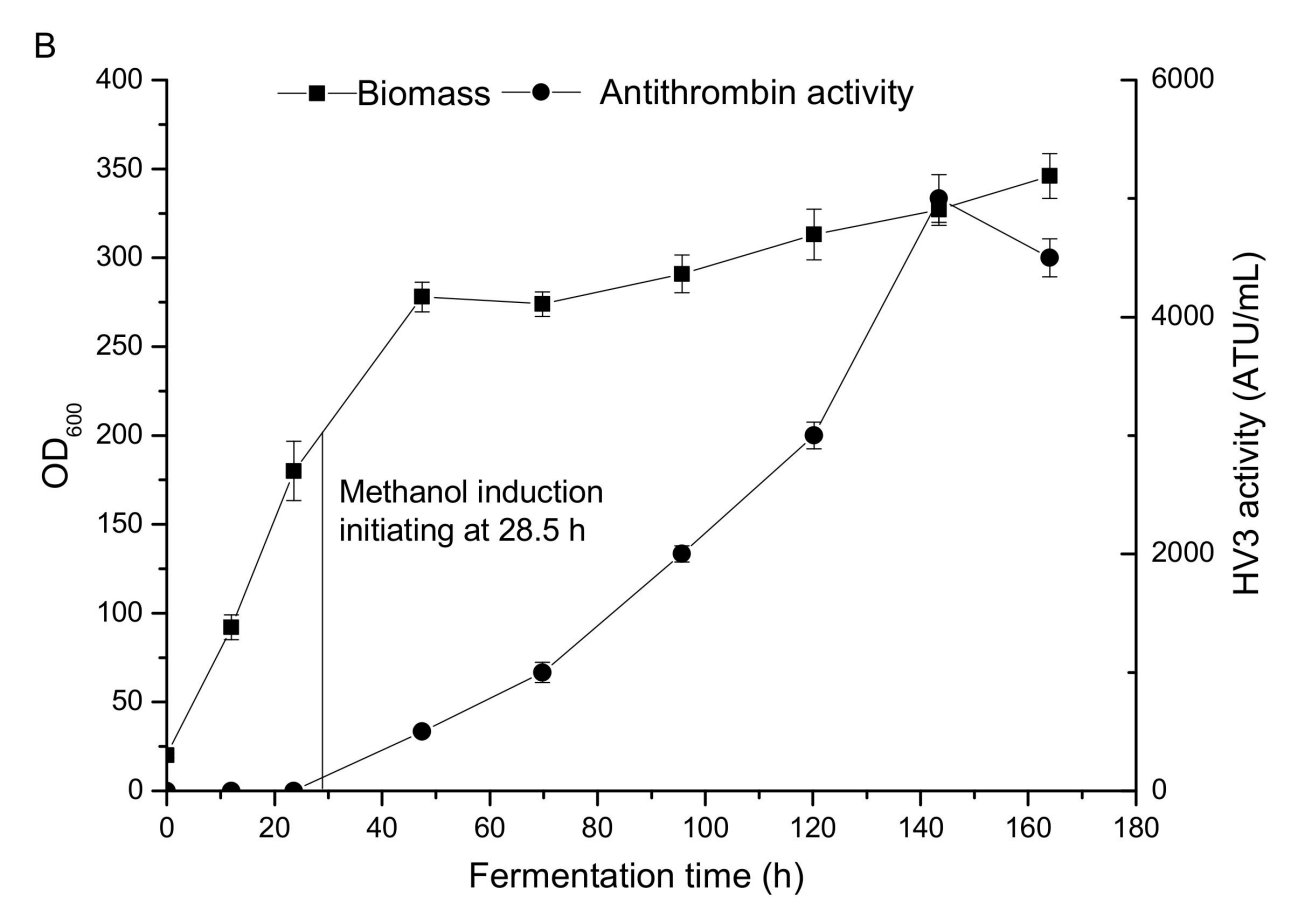
HV3 expression in the recombinant *P. pastoris* GS115/pPIC9K-*hv3* (**A**) and its fermentation curve in 5 L fermenter (**B**).

The sequencing result indicated the successful recombination of gene *hv3* in *P. pastoris* GS115/pPIC9K-*hv3* (data not shown). To further validate the successful translation of *hv3* and its antithrombin activity, we cultivated *P. pastoris* GS115/pPIC9K-*hv3* and *P. pastoris* GS115/pPIC9K in BMMY medium in which methanol served as the sole carbon source and an inducer for *hv3* expression (Figure 1A). The antithrombin activity in the broth of *P. pastoris* GS115/pPIC9K-*hv3* reached the maximum value of 25 ATU/mL, while the fermentation liquor of *P. pastoris* GS115/pPIC9K did not show any antithrombin activity.

Fermentation of *P. pastoris* GS115/pPIC9K-*hv3* was conducted in a 5 L fermenter to further improve the production of HV3, and the highest antithrombin activity reached 5000 ATU/mL after 143.4 h of cultivation. Additionally, the synthesis of HV3 was almost synchronous with cell growth during the methanol induction phase (Figure 1B). In order to obtain a deep insight on the transcriptional change after carbon source switching from glycerol to methanol, a comparative transcriptomic study of three timepoint samples (collected at 24 h, before the methanol induction initiation, designated as BI; at 96 h and 132 h, named as PI1 and PI2, respectively) was performed (Figure 1B).

### 3.2. Overall Evaluation of RNA-Seq Data

The total RNA of sample BI, PI1, and PI2 were extracted and evaluated in triplicates, named as BI_1, BI_2, and BI_3; PI1_1, PI1_2, and PI1_3; PI2_1, PI2_2, and PI2_3, respectively. Then equal amounts of total RNA of each sample was employed for Illumina sequencing, and the overall quality of the high-throughput sequencing data was evaluated and shown in Table S1. The proportion of clean reads for each sample was approximately above 94%. Phred quality score (Q score) was employed to measure the base calling accuracy during sequencing [41]. Q_30_ is equivalent to the probability of an error base call 1 in 1000 times (Table S1), meaning the base calling accuracy is 99.9%. Similarly, Q_20_ stands for the 99% accuracy of base calling. The proportion of Q_20_ was larger than 95%, and the proportion of Q_30_ was no less than 88.92%. Therefore, the quality of sequencing data was good enough for the following sequence analysis. The percentage of reads uniquely mapped to the reference genome was distributed in the range from 92.28% to 94.42% (Table S2), indicating the validity of the employed reference genome. By comparing the gene expression level of sample BI with that of PI1 and PI2, we could conclude that the proportion of high abundant mRNA was increased in the recombinant *P. pastoris* during the methanol induction phase (Table S3). In order to check the sample quality of biological replicates for each sample, the Pearson correlation coefficients R^2^ were calculated. The results showed that the R^2^ of replicates for each group was above 0.997, indicating no significant difference between the biological replicates of each sample (Figure S1).

### 3.3. Overview of Transcriptomic Analysis

1239 differently expressed genes (DEGs), including 690 up-regulated and 549 down-regulated genes, were determined in PI1 in comparison to BI (Figure S2A). It indicated the significant change of gene expression pattern in *P. pastoris* after the carbon source switching from glycerol to methanol. When being continuously cultivated for 47 h, the number of DEGs in PI2 became 1365—696 up-regulated genes, 669 down-regulated ones (Figure S2B). Compared with PI1, 14 genes were up-regulated in PI2, while the down-regulated genes were 107 (Figure S2C). DEGs was clustered, and main gene expression pattern of sample BI was almost completely contrary with that in PI1 and PI2 (Figure S3). The gene clusters with the low expression level (blue area) in BI almost entirely became up-regulated (red part) in PI1 and PI2.

### 3.4. GO (Gene Ontology) Enrichment Analysis of DEGs

The functional distributions of those DEGs of three groups (group A: PI1 versus BI; B: PI2 versus BI; C: PI2 versus PI1) were annotated and analyzed. The abundant genes were categorized into 30 major functional terms according to the GO category, and metabolic process, catalytic activity, single-organism metabolic process, oxidoreductase activity, and small molecule metabolic process were the top five in group A (Figure S4A). DEGs belonging to the category of catalytic activity, single-organism metabolic process, and oxidoreductase activity were the most abundant parts in group B (Figure S4B). However, the most abundant categories in group C were membrane and related parts belonging to cellular components, indicating cellular structure alteration as the proceeding of methanol induction (Figure S4C).

### 3.5. Analysis of DEGs Involved in Central Carbon Metabolic Pathway

**Figure 2:**
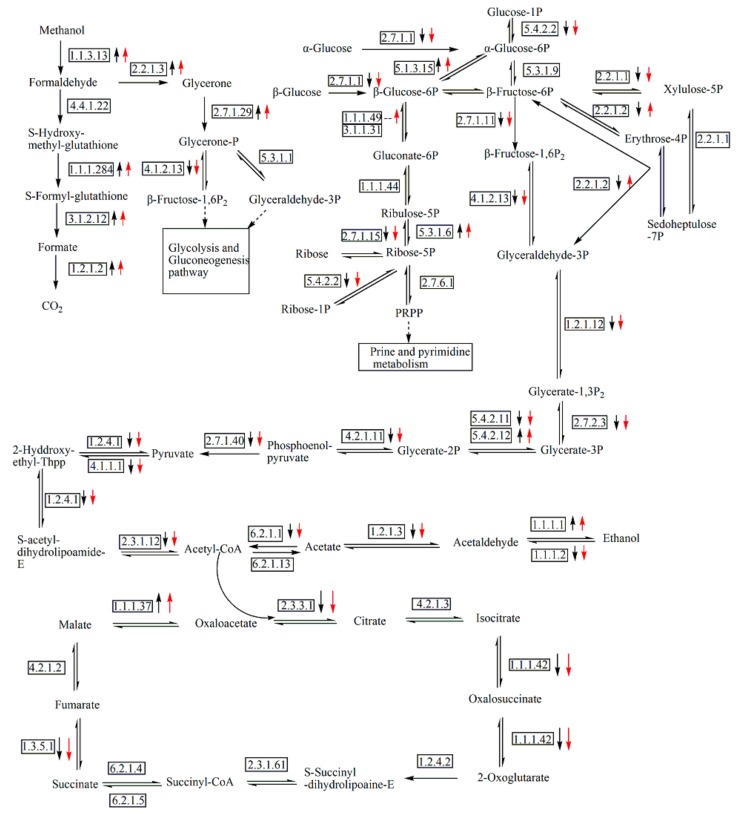
Overview of DEGs involved in central carbon metabolic pathway in PI1 and PI2 in comparison to BI. Up-regulated or down-regulated genes (*p*_adj_ < 0.01) are depicted with up or down arrow (black arrow stands for PI1, red arrow indicates PI2), respectively. A dash line indicates no significant change in comparison to BI.

The central carbon metabolic pathway and relative enzymes were sketched in Figure 2 by referring to the KEGG database. Genes encoding enzymes EC1.1.3.13, EC4.4.1.22, EC1.1.1.284, EC3.1.2.12, EC1.2.1.2, EC2.7.1.29, and EC2.2.1.3 in PI1 and PI2, were greatly up-regulated during the methanol induction phase, and were responsible for the conversion of methanol to CO_2_ and glycerone-P (Table 1). In addition, genes PAS_chr1-4_0042 and PAS_chr4_0815 encoding glucose-6-phosphate 1-epimerase (EC5.1.3.15) and mitochondrial malate dehydrogenase were also up-regulated significantly in PI1 and PI2 (Figure 2, Table 1). Reaction from glycerate-2P to glycerate -3P was catalyzed by two enzymes, EC5.4.2.11 (PAS_chr3_0826) and EC 5.4.2.12 (PAS_chr2-2_0177). And the expression pattern of the above two genes which located on different chromosomes was different, one was up-regulated, the other was down-regulated. Similar phenomenon was also observed in the reaction from acetaldehyde to ethanol (Figure 2).

Glycerone-P converted from methanol was transformed into β-fructose-1,6 P_2_ and glyceraldehyde-3P at the catalysis of fructose 1,6-bisphosphate aldolase (EC4.1.2.13) and glyceraldehyde-3-phosphate aldose-ketose-isomerase (EC5.3.1.1). Those two intermediates entered into the glycolysis/gluconeogenesis and citrate cycle pathways. However, most of the genes involved in those pathways were down-regulated in PI1 and PI2, especially for those encoding key regulatory or rate-limiting enzymes, including hexokinase-2 (EC2.7.1.1), phosphofructokinase (EC2.7.1.11), pyruvate kinase (EC2.7.1.40), citrate synthase (EC2.3.3.1), and isocitrate dehydrogenase (EC1.1.1.42) (Figure 2, Table 1). As for the comparison of gene expression between PI1 and PI2, only the log_2_(fold change) of PAS_chr4_0624 and PAS_chr1-4_0561 (EC2.7.1.1) was over 1, and the expression of other genes involved in the central carbon metabolic pathway did not show significant variation based on our screening criteria (Figure 2).

### 3.6. Carbon Source Switching from Glycerol to Methanol Resulted in a Significant Slowdown of Energy Metabolism

**Figure 3:**
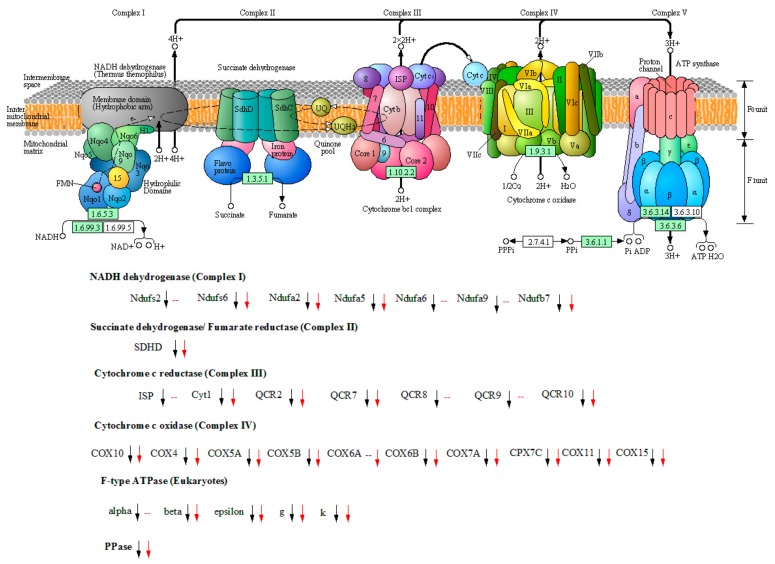
Overview of DEGs participating in the mitochondrial oxidative phosphorylation process in PI1 and PI2 in comparison to BI. Up-regulated or down-regulated genes in comparison to BI (*p*_adj_ < 0.01) are depicted with up or down arrow (black arrow stands for sample PI1, red arrow indicates PI2), respectively. A dash line indicates no significant change in comparison to BI. The picture was cited from KEGG database during transcriptomic analysis.

Nearly all the detected genes encoding proteins distributed in almost all units of oxidative phosphorylation process were down-regulated in PI1 and PI2 in comparison to BI, except for few genes whose expression level didn’t change significantly in PI2, such as PAS_chr3_1188 (encoding Ndufa9), PAS_chr3_0808 (Ndufs2), PAS_chr4_0535 (Ndufa6), PAS_chr2-1_0850 (ISP), PAS_chr1-4_0313 (QCR8), PAS_chr4_0520 (QCR9), and PAS_chr3_0576 (α) (Figure 3 and Table 2). PAS_chr1-3_0070 encoding mitochondrial inorganic pyrophosphatase, which converts diphosphate to phosphate for ATP production at the catalysis of ATPase, was also down-regulated in PI1 and PI2. Our results indicated that the energy metabolism in the recombinant *P. pastoris* declined comprehensively. Moreover, the expression level of genes involved in energy metabolism in PI2 did not show significant difference with that in PI1, according to our screening criteria (Table S5).

### 3.7. Expression Analysis of Genes Involved in the Biogenesis of Peroxisome and Peroxisomal Proteins

**Figure 4:**
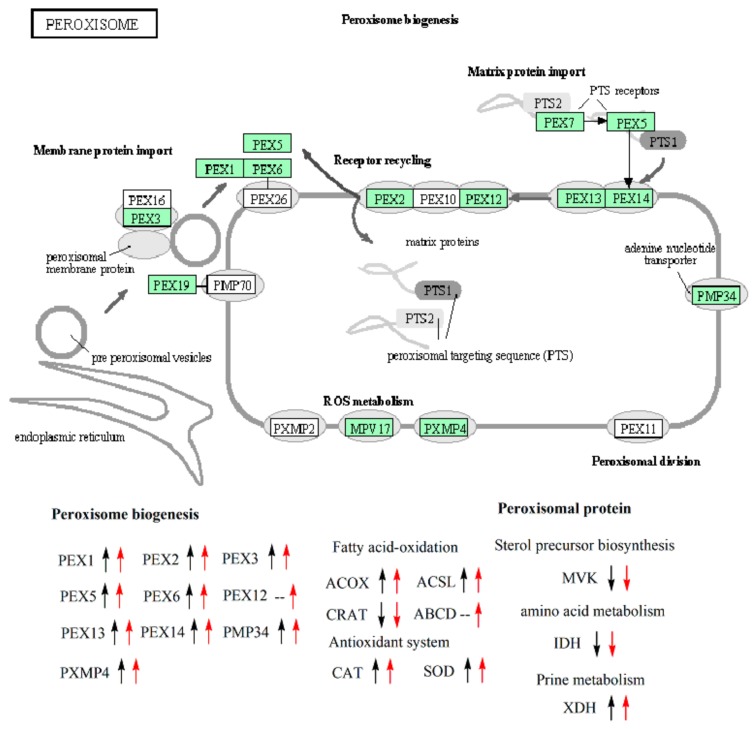
Overview of DEGs in peroxisome in PI1 and PI2 in comparison to BI. Up-regulated or down-regulated genes in comparison to BI (*p*_adj_ < 0.01) are depicted with up or down arrow (black arrow stands for sample PI1, red arrow indicates PI2), respectively. A dash line indicates no significant change in comparison to BI. The picture was cited from KEGG database during transcriptomic analysis.

Nearly all the detected genes involved in peroxisome biogenesis were up-regulated in PI1 and PI2, except for PAS_chr4_0759 (encoding PEX12) whose expression level did not change significantly in PI1 in comparison to BI (Figure 4, Table 3). Some genes encoding peroxisomal proteins were also up-regulated in PI1 and PI2 in comparison to BI. PAS_chr2-2_0131 and PAS_chr4_0786 encoding the important and representative antioxidant enzymes, such as catalase (CAT) and superoxide dismutase (SOD), were remarkably up-regulated in both PI1 and PI2. On the contrary, PAS_chr1-4_0074 encoding outer mitochondrial carnitine acetyltransferase (CRAT), an enzyme involved in the fatty acid oxidation process in peroxisome, was down-regulated. But the other three genes (PAS_chr1-4_0538, PAS_chr4_0352, and PAS_chr2-1_0785) encoding fatty-acyl coenzyme A oxidase (ACOX) and long chain fatty acyl-CoA synthetase (ACSL) were up-regulated in this process. Besides, PAS_chr2-2_0272 encoding peroxisomal ATP-binding cassette transporter complex was also up-regulated in PI2, not in PI1. Genes encoding mevalonate kinase (MVK), isocitrate dehydrogenase (IDH), xanthine dehydrogenase (XDH) involved in sterol precursor biosynthesis, amino acid metabolism, and the purine metabolism process, respectively, were all down-regulated in both PI1and PI2. We also found that the expression of gene PAS_chr2-2_0207 encoding PEX13, an integral peroxisomal membrane protein, in PI2 increased significantly in comparison to PI1 according to our screening criteria (Table S5).

### 3.8. Transcriptomic Analysis of Genes Participating in the Protein Production and Degradation Processes

**Figure 5:**
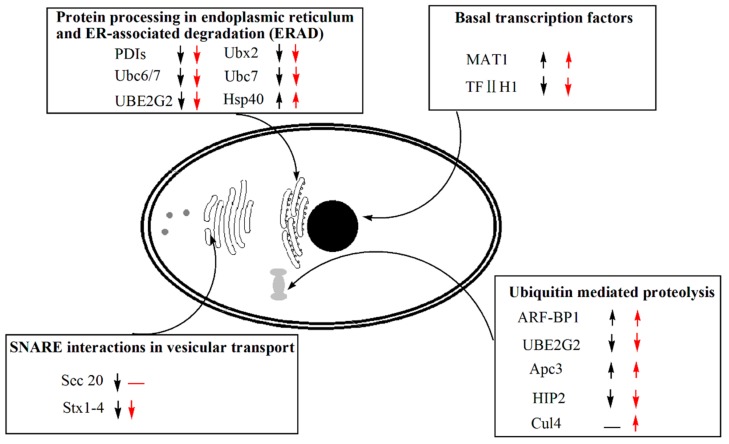
Overview of DEGs in peroxisome in PI1 and PI2 in comparison to BI. Up-regulated or down-regulated genes in comparison to BI (*p*_adj_ < 0.01) are depicted with up or down arrow (black arrow stands for sample PI1, red arrow indicates PI2), respectively. A dash line indicates no significant change in comparison to BI.

The expression level analysis of genes regarding to the protein production and degradation processes is vital and necessary for getting a deep understanding of the recombinant protein/peptide production in *P. pastoris*. We focused on four closely related aspects of the protein production and degradation, including basal transcription factors, protein processing in endoplasmic reticulum and ER-associated degradation (ERAD), SNARE interaction in vesicular transport, and ubiquitin mediated proteolysis (Figure 5). Only a few DEGs were detected in both PI1 and PI2, in comparison to BI, during methanol induction stage (Table 4, Figure 5). The expression level of PAS_chr4_0204 and PAS_chr4_0745, which encode transcription factors MAT1 and TFIIH1, varied in contrast in PI1 and PI2 in comparison to BI—the former one increased, but the latter one decreased.

As regards to SNARE interaction in vesicular transport in Golgi body, only PAS_chr1-4_0294 encoding Stx1-4 was down-regulated in both PI1 and PI2. PAS_chr1-4_0462 (Table 4) was solely down-regulated in PI1, not in PI2. PAS_chr2-2_0066 encoding Hsp40 was the only one with an increased expression level, but the others were all down-regulated. The expression level of genes involved in ubiquitin mediated proteolysis also varied in PI1 and PI2 in comparison to BI. PAS_chr3_0044 and PAS_chr1-3_0148, which encode E3 ubiquitin ligase (ARF-BP1) and ubiquitin conjugating enzyme (Apc3) respectively, were remarkably up-regulated in both PI1 and PI2. However, PAS_chr3_0924 and PAS_chr4_0429 encoding ubiquitin-conjugating enzyme (UBE2G2 and HIP2) was down-regulated. Besides, PAS_chr1-4_0609 encoding cullin, a structural protein of SCF complexes, a multi-protein E3 ubiquitin ligase complex, was also up-regulated in PI2, not in PI1. The expression level of genes involved in protein production and degradation processes in PI2 did not show a significant difference with that of PI1 (Table S5).

### 3.9. Overall Analysis of Genes Involved in Amino Acid Biosynthesis

The proportion of up-regulated genes participating in the amino acid biosynthesis in PI1 and PI2 was very small, merely including genes encoding enzymes EC5.4.99.5, EC5.3.1.6, EC3.5.3.1, EC2.3.1.1, and EC2.7.2.11 (Figure S5, Table S4). Besides, PAS_chr4_0645, PAS_chr4_0974, PAS_chr1-4_0489, and PAS_chr2-2_0288 encoding enzymes EC2.6.1.11, EC2.6.1.1, EC4.4.1.1, and EC3.5.3.1 were only up-regulated in PI2 other than PI1. In general, most of the genes involved in the amino acid biosynthetic pathways were down-regulated. Moreover, almost all genes participating in the biosynthetic pathways of serine, glycine, alanine, glutamate, tyrosine, and cysteine were detected, and those were all down-regulated (Figure S5). The expression level of PAS_chr4_0645 (EC2.6.1.11) and PAS_chr1-4_0160 (EC3.6.1.31/3.5.4.19/1.1.1.23) participating in the arginine and histidine biosynthesis process decreased further in PI2 in comparison to PI1 (Table S5). In conclusion, our transcriptomic study indicated the comprehensive decline of amino acid biosynthesis in the recombinant *P. pastoris* when being cultivating in the methanol medium.

## 4. Discussion

The methylotrophic yeast *P. pastoris* (syn. *Komagataella* spp.) has become one of the most important and widely-used expression systems for producing heterologous proteins, especially after the release of its genome sequence in 2009 [42]. High secretory capacity, a strong methanol inducible promoter and the existence of a glycosylation pathway are three attractive advantages over other eukaryotic expression systems, which allows a broad spectrum of recombinant proteins to be produced in this host, especially for those from higher eukaryotic cells [24,43,44,45]. However, some problems of this system should not be ignored. The production of heterologously expressed protein is improved by increasing the gene copy number. But an optimal gene copy number seems to exist for each recombinant protein [46,47]. The clonal variation still seems to be the major bottleneck for the industrial application of this host system because hundreds of clones per phenotype need to be screened to get the highest secretor [26,27]. The productivity would be varied even if the recombinants own the same gene copy number. However, this problem cannot be attributed to only one level (transcriptional or translational level) [26]. We have often encountered similar problems, and the same operation of this host for different kinds of genes has usually brought in different results. The chance of successful expression and a high production of heterologous proteins have usually been very low. Moreover, the employment of two carbon sources (glucose or glycerol for cell growth and methanol for protein expression) and switching from one to the other at a precise or proper time point was difficult to realized [24,28]. Fortunately, we have the recombinant *P. pastoris* GS115/pPIC9K-*hv3* which can produce HV3 efficiently, and have also found that the time for switching methanol induction has a profound influence on the final yield of HV3—an early or late time was not appropriate (data not shown). Moreover, we found that the function of methanol was not only in inducing exogenous genes expression, but in supporting cell growth as the carbon source. In a word, we found that the biosynthesis of HV3 was actually closely related to the growth of *P. pastoris*. If the cells hardly grew during the late methanol induction stage (Figure 1B), the biosynthesis of HV3 also stopped. Shan Zhou et al. found that the specific growth rate and methanol concentration also influenced the degradation rate of hirudin produced by *P. pastoris*. The maximum specific hirudin production rate was achieved when a specific growth rate was maintained at 0.02 h^−1^ [48]. In order to get a full insight on the effects of methanol on the cell growth and expression of heterologous proteins in recombinant *P. pastoris*, and develop an appropriate strategy to further improve the production of HV3, we investigated the whole cellular gene expression of samples collected before and after methanol induction by transcriptomic method. 

AOX encoded by gene *AOX1* is the first and most important enzyme in initializing the methanol utilization pathway in methylotrophic yeast *P. pastoris*. Given that the promoter of *AOX1* is strong-methanol inducible, the expression vector for heterologous protein is usually constructed by using the promoter sequence, and the induction of transcription is easily realized by using methanol as an inducer [24]. We adopted the same strategy for producing HV3 in the recombinant *P. pastoris*. The expression of PAS_chr4_0821, encoding alcohol oxidase (EC 1.1.3.13), was greatly up-regulated in *P. pastoris* GS115/pPIC9K-*hv3* during the methanol induction phase (Table 1, Figure 2, PI1 vs. BI, PI2 vs. BI), the log_2_ (fold change) reached 6.5268 and 6.6696 in sample PI1 and PI2 in comparison to BI, respectively. Kim et al. investigated the regulation of *AOX1* promoter activity and peroxisome biogenesis by visualizing the expression and localization of green fluorescent protein (GFP)-fused proteins under different fermentation processes, including a methanol-limited condition at normoxy (ML), switched feeding of carbon sources (glucose and methanol) under carbon-limited condition at normoxy (SML), and an oxygen-limited (OL) condition. The experiment results indicated that the yield of a foreign protein was highly dependent on the methanol consumption rate influenced by the availability of methanol and oxygen molecules [49]. But we could not agree with this viewpoint completely. Excessive methanol in the broth is harmful to the yeast cell [50]. Afterall, the production of heterologous protein in *P. pastoris* is a quite complex process, which is related to transcription, translation, protein transport and export, protein modification, excretion processes, and so on. Once the normal physiological state is affected by excessive toxic methanol, the productivity of protein would probably decrease in cells. Therefore, the key point for improving methanol consumption rate should be placed on the enhancement of methanol assimilation ability by *P. pastoris*, not on the strategy for the fed-batch methanol supplementation. 

How can the methanol assimilation rate be fundamentally improved? To address this question, it is necessary to view the overall expression variation of genes involved in the central carbon metabolism in *P. pastoris* during the methanol induction phase (Figure 2). AOX (EC1.1.3.13, Figure 2), dihydroxyacetone synthase (EC2.2.1.3, Figure 2, Table 1), and catalase (CAT, Table 3, Figure 4) were the three essential enzymes responsible for the conversion of methanol to non-toxic intermediates which subsequently entered into the central carbon metabolism pathway and supported the biomass production [51,52]. Kim et al. investigated the effect of methanol in inducing the biogenesis of peroxisome in the recombinant *P. pastoris*, which showed a high dependency on methanol consumption [49]. The transcriptomic results of our study also supported this viewpoint, and the detected DEGs involved in peroxisome biogenesis process were all up-regulated during the methanol induction phase (Figure 4). But the consumption of methanol should not merely depend on the above three enzymes present in peroxisome (Figure 2). Actually, three branched pathways for methanol metabolism exist in yeast *P. pastoris*. In one branched pathway, methanol was finally converted into CO_2_ with the catalysis of a series of enzymes, including EC1.1.1.284, EC3.1.2.12, and EC1.2.1.2, whose encoding genes were significantly up-regulated in the recombinant *P. pastoris* during the methanol induction phase. Glycerone-P and glyceraldehyde-3P which were converted from formaldehyde subsequently entered into the glycolysis/gluconeogenesis pathway and TCA cycle. However, the genes involved in these pathways, especially those encoding rate-limiting enzymes, were nearly all remarkably down-regulated. Obviously, this was not favorable for biomass production as the intermediates in these pathways were the main materials for protein, lipid, and polysaccharide biosyntheses. These results also coincided with our fermentation study which showed that the cells obviously grew slowly during the methanol induction phase (Figure 1B). Besides, the expression level of those genes did not increase significantly with the induction time (PI2 vs. PI1, Table S5). Therefore, if we want to improve the methanol consumption rate and the productivity of foreign proteins, the overall enhancement of the central carbon metabolism, including glycolysis/gluconeogenesis, TCA cycle, should be feasible and effective. Joel Jordà et al. tried using a mixed carbon source (methanol and glucose) in the induction phase, and found a significant redistribution of carbon fluxes in the central carbon metabolism, reflecting not only in increased glycolytic, TCA cycle, and NADH regeneration fluxes but also as higher methanol dissimilation rates [53]. Hence this study also backed our proposal that increased carbon fluxes from methanol to glycolysis, TCA cycle and so on would not only benefit the growth of the recombinant *P. pastoris*, but also facilitated the methanol consumption and foreign protein production. Thus, a balanced central carbon metabolism should be focused on for the production of heterologous protein in *P. pastoris*.

Research focusing on the energy metabolism comparison in *P. pastoris* cultivated using glycerol or methanol as a carbon source are still very few. Joel Jordà et al. asserted that the oxidation of methanol requires large amounts of ATP, while Veeresh et al. held that the oxidation process from GSCH_2_OH to formate, and the final product CO_2_, actually released large amounts of energy in the form of NADH which eventually entered into the oxidative phosphorylation process [24,53]. Our results showed that the genes involved in the oxidative phosphorylation process were actually down-regulated significantly (Figure 3, Table 2). As a result, ATP production should decline in the recombinant *P. pastoris* during the methanol induction stage in comparison to that cultivated in glycerol medium (PI1 vs. BI, PI2 vs. BI). This phenomenon could be attributed to the down-regulation of genes participating in TCA cycle and glycolysis (Figure 2). As a result, the total amount of NADH entering into the electron transfer chain decreased accordingly due to the slow-down of the central carbon metabolism. Our conclusion is that the amounts of mitochondrial respiratory chain and the ATP production rate declined in the recombinant *P. pastoris* after carbon source switching from glycerol to methanol. Moreover, this situation did not change with the methanol induction process (Figure 3, Table 2, PI2 vs. BI). This could also explain the slowdown of cell growth during the methanol induction phase due to the decrease of ATP production.

The expression of genes related to protein production and degradation were also analyzed (Figure 4, Table 4). Only the expression of two transcription factors varied significantly in *P. pastoris* during the methanol induction phase. Moreover, two genes encoding Sec20 and Stx 1-4, which were responsible for secreting foreign proteins involved in SNARE interaction in the vesicular transport pathway, were down-regulated in *P. pastoris*. Besides we also investigated the expression level of genes involved in protein processing in the endoplasmic reticulum and ER-associated degradation pathway (ERAD). However, most of them were down-regulated. Yeast and other eukaryotic cells would activate the Unfolded Protein Response (UPR) pathway if the unfolded or misfolded proteins accumulated in ER lumen by inducing the expression of genes involved in protein folding and ERAD. The misfolded secretory proteins will be retro-translocated to the cytoplasmic side of the ER, poly-ubiquitinated, and delivered to the proteasome for degradation [54]. Our results showed that the ERAD pathway was not up-regulated and we inferred that the possible reasons were the low molecular weight of HV3 and its relatively simple conformation. As a result, the rate for misfolding would be very low. Besides, gene encoding HSP40, a protein chaperon, was also up-regulated in *P. pastoris* during the methanol induction stage (Table 4), and it possibly played an important role in HV3 folding. Most of the genes involved in amino acids biosynthesis were also down-regulated (Figure S5, Table S4). According to the above results, we proposed that the general cellular protein abundance should be reduced even though HV3 was successfully produced by *P. pastoris* GS115/pPIC9K-*hv3* during the methanol induction phase. Hence, little stress of HV3 synthesis and protein secretion occurred in this recombinant yeast but there is still a large space for improving the yield of this recombinant peptide by this strain.

Previous studies also illustrated the profiles of gene expression in *P. pastoris* cultivated in medium with different carbon sources (glucose, glycerol, or methanol) [55,56]. Two to three folds of genes were highly and selectively expressed in *P. pastoris* cultivated in methanol medium in comparison to those in glucose- or glycerol-based cultivation. However, genes involved in central metabolism, amino acid metabolism, lipid metabolism, and transport process were all similarly highly-expressed in *P. pastoris* no matter under which type of cultivation model (glucose-, glycerol, or methanol cultivation). But the genes related to peroxisomal, protein folding, and stress response were more enriched during cultivation in methanol [55]. Liang et al. (2012) also investigated the variation of gene expression in *P. pastoris* cultivated in methanol medium in comparision to that in glycerol medium. The genes involved in methanol metabolic pathway and proteasome were significantly up-regulated with the methanol induction [56]. However, the above studies all collected the batch culture of *P. pastoris* in methanol or glycerol to conduct the comparative transcriptomic analysis. We assert that the growth characteristics of *P. pastoris* in those two mediums are totally different. Thus, a comparison of gene expression in those cultures collected at the same time was not very scientific and rational. Methanol has been used as a carbon source to induce the expression of heterologous protein in *P. pastoris* for decades [57]. Nowadays, the two carbon source cultivation strategy has been widely applied for the production of heterologous proteins in *P. pastoris*, and the shift from glycerol to methanol was an important process [52,56]. Hence, it would be more valuable for guiding the host cell engineering for improved heterologous protein production by investigating and analyzing the influence of a carbon source switch on the gene expression in *P. pastoris*. Actually, our experiment results were partially consistent with the previous studies, such as an enhanced methanol utilization pathway during the methanol induction phase. Moreover, we almost analyzed the expression of every gene involved in the central carbon metabolism, amnio acid metabolic, mitochondrial oxidative phosphorylation process, and so on. Our research outcome presents a promising way to improve the heterologous protein production by enhancing the central carbon metabolism which will eventually improve the utilization of methanol. This would be beneficial for both the cell growth of *P. pastoris* and heterologous protein production due to the accelerated methanol consumption rate. 

In conclusion, we successfully constructed a recombinant *P. pastoris* GS115/pPIC9K-*hv3* for producing HV3, and the antithrombin activity in the fermentation broth reached 5000 ATU/mL. We observed that the biosynthesis of HV3 also became slowed down when the methanol consumption and cell growth rate declined. Our preliminary transcriptomic results showed that glycerate-P converted from methanol enters into the central carbon metabolism, which together with energy metabolism and amino acid biosynthesis, were almost all down-regulated completely in *P. pastoris* GS115/pPIC9K-*hv3* during the methanol induction phase. Thus, we propose that the up-regulation of glycolysis, pentose phosphate pathway, TCA cycle, energy metabolism, amino acid, and lipid metabolism should be beneficial for both improving methanol consumption and HV3 production in *P. pastoris* GS115/pPIC9K*-hv3*. Besides, genes involved in EARD, the ubiquitin mediated proteolysis pathway and proteasome were not up-regulated significantly, indicating that the expression of HV3 did not give rise to foreign protein stress and a huge potential still exists for improving the production of HV3 in *P. pastoris* GS115/pPIC9K-*hv3*.

## Figures and Tables

**Table 1:** Information of differentially expressed genes (DEGs) involved in the central carbon metabolic pathway in PI1 and PI2 in comparison to BI.

EC No.	Gene Name	Gene ID	Gene Description	Log_2_ (Fold Change)/PI1 vs. BI	*p*_adj_/PI1 vs. BI	Log_2_ (Fold change)/PI2 vs. BI	*p*_adj_/PI2 vs. BI
6.2.1.1	PAS_chr3_0403	8199535	Acetyl-coA synthetase isoform	−4.2415	0	−4.2022	0
2.7.1.1	PAS_chr1-4_0561	8197692	Hexokinase-2	−2.4875	0	−3.6289	0
1.2.1.3	PAS_chr3_0987	8199733	Mitochondrial aldehyde dehydrogenase	−2.2758	1.95 × 10^−237^	−1.5456	5.74 × 10^−131^
2.7.2.3	PAS_chr1-4_0292	8197742	3-phosphoglycerate kinase	−2.1073	0	−2.8743	0
4.2.1.11	PAS_chr3_0082	8199366	Enolase	−1.9763	7.89 × 10^−307^	−2.5303	0
1.1.1.2	PAS_chr4_0576	8200841	NADPH-dependent medium chain alcohol dehydrogenase	−1.9642	7.25 × 10^−181^	−1.8923	7.45 × 10^−190^
4.1.2.13	PAS_chr1-1_0072	8197200	Fructose 1,6-bisphosphate aldolase	−1.9342	3.70 × 10^−282^	−2.0982	0
1.2.1.12	PAS_chr2-1_0437	8198905	Glyceraldehyde-3-phosphate dehydrogenase	−1.9318	1.00 × 10^−134^	−2.3868	4.91 × 10^−185^
5.4.2.11	PAS_chr3_0826	8200319	Tetrameric phosphoglycerate mutase	−1.8358	0	−2.0814	0
5.4.2.2	PAS_chr1-4_0264	8197873	Phosphoglucomutase	−1.7789	0	−2.0539	0
2.7.1.11	PAS_chr2-1_0402	8198870	Phosphofructokinase involved in glycolysis	−1.759	0	−2.711	0
2.7.1.15	PAS_chr3_0604	8199807	Putative ribokinase	−1.7244	0	−2.3145	0
4.1.1.1	PAS_chr3_0188	8199939	Major of three pyruvate decarboxylase isozymes	−1.5442	4.26 × 10^−187^	−2.4152	0
1.2.4.1	PAS_chr1-4_0593	8197723	E1 β subunit of the pyruvate dehydrogenase (PDH) complex	−1.5393	2.17 × 10^−131^	−1.676	3.23 × 10^−157^
2.7.1.11	PAS_chr1-4_0047	8196884	β subunit of heterooctameric phosphofructokinase	−1.4239	0	−1.8729	0
2.7.1.40	PAS_chr2-1_0769	8198046	Pyruvate kinase	−1.3692	0	−1.9631	0
2.2.1.1	PAS_chr1-4_0150	8197134	Transketolase	−1.3666	5.53 × 10^−118^	−1.3961	2.01 × 10^−134^
5.4.2.11	PAS_chr3_0693	8200393	Tetrameric phosphoglycerate mutase	−1.0681	4.40 × 10^−55^	−1.2688	2.30 × 10^−74^
2.2.1.2	PAS_chr2-2_0337	8198237	Transaldolase	−1.0501	1.28 × 10^−44^	--	--
2.3.1.12	PAS_chr1-1_0050	8197567	Dihydrolipoamide acetyltransferase component (E2) of pyruvate dehydrogenase complex	−1.028	1.03 × 10^−187^	−1.2588	3.15 × 10^−240^
5.3.1.6	PAS_chr4_0213	8200884	Ribose-5-phosphate ketol-isomerase	1.8505	0	2.1047	4.199 × 10^−314^
2.7.1.29	PAS_chr3_0841	8200330	Dihydroxyacetone kinase	2.1754	0	2.3308	0
5.1.3.15	PAS_chr1-4_0042	8196879	Glucose-6-phosphate 1-epimerase	2.2902	0	2.6352	0
5.4.2.12	PAS_chr2-2_0177	8198274	Putative protein of unknown function	2.419	0	2.267	0
1.1.1.284	PAS_chr3_1028	8199772	S-(hydroxymethyl)glutathione dehydrogenase	3.401	0	3.714	0
1.1.1.1	PAS_chr3_1028	8199772	S-(hydroxymethyl)glutathione dehydrogenase	3.401	0	3.714	0
3.1.2.12	PAS_chr3_0867	8200354	Esterase function as an S-formylglutathione hydrolase	3.9897	0	4.2466	0
1.1.3.13	PAS_chr4_0821	8201223	Alcohol oxidase	6.5268	0	6.6696	0
5.3.1.6	PAS_chr4_0212	8200883	Ribose-5-phosphate ketol-isomerase	6.5546	0	6.9045	0
2.2.1.3	PAS_chr3_0832	8199663	Transketolase, dihydroxyacetone synthase	6.7656	0	6.9536	0
1.2.1.2	PAS_chr3_0932	8200284	NAD (+)-dependent formate dehydrogenase	7.1213	0	7.5608	0
2.2.1.3	PAS_chr3_0834	8200324	Transketolase	7.8929	0	8.1561	0
1.1.1.49	PAS_chr2-1_0308	8198996	Glucose-6-phosphate dehydrogenase (G6PD)	--	--	1.1109	2.86 × 10^−271^
2.2.1.2	PAS_chr2-2_0338	8198238	Transaldolase	--	--	6.8356	0
1.1.1.42	PAS_chr2-1_0580	8198933	Cytosolic NADP-specific isocitrate dehydrogenase	−2.9882	4.16 × 10^−277^	−3.1716	2.877 × 10^−311^
1.2.4.1	PAS_chr1-4_0593	8197723	E1 β subunit of the pyruvate dehydrogenase (PDH) complex	−1.5393	2.17 × 10^−131^	−1.676	3.23 × 10^−157^
1.3.5.1	PAS_chr3_0424	8199556	Membrane anchor subunit of succinate dehydrogenase	−1.3199	3.86 × 10^−139^	−1.4013	1.19 × 10^−148^
2.3.3.1	PAS_chr1-1_0475	8197246	Citrate synthase	−1.3184	1.47 × 10^−121^	−1.3199	4.41 × 10^−131^
1.1.1.42	PAS_chr1-1_0233	8196735	Mitochondrial NADP-specific isocitrate dehydrogenase	−1.0698	4.44 × 10^−202^	−1.0284	4.63 × 10^−179^
2.3.1.12	PAS_chr1-1_0050	8197567	Dihydrolipoamide acetyltransferase component (E2) of pyruvate dehydrogenase complex	−1.028	1.03 × 10^−187^	−1.2588	3.15 × 10^−240^
1.1.1.37	PAS_chr4_0815	8201217	Mitochondrial malate dehydrogenase	1.381	0	1.7676	0
1.2.4.1	PAS_chr2-2_0294	8198194	E1 α subunit of the pyruvate dehydrogenase (PDH) complex	--	--	−1.006	1.59 × 10^−228^

A dash line indicates no significant change in comparison to BI.

**Table 2:** List of DEGs involved in mitochondrial oxidative phosphorylation process in PI1 and PI2 in comparison to BI.

Protein Name	Gene Name	Gene ID	Gene Description	Log_2_ (Fold Change)/PI1 vs. BI	*p*_adj_/PI1 vs. BI	Log_2_ (Fold Change)/PI2 vs. BI	*p*_adj_/PI2 vs. BI
Ndufb7	PAS_chr1-1_0172	8196676	Hypothetical protein	−1.0432	1.07 × 10^−89^	−1.4134	1.01 × 10^−108^
Ndufa2	PAS_chr1-4_0575	8197446	NADH-ubiquinone oxidoreductase	−1.2501	4.90 × 10^−108^	−1.2525	6.41 × 10^−106^
Ndufa5	PAS_chr1-4_0371	8197883	Hypothetical protein	−1.2085	1.92 × 10^−191^	−1.1128	1.15 × 10^−158^
Ndufs6	PAS_chr2-2_0235	8198642	Hypothetical protein	−1.1401	1.73 × 10^−156^	−1.1829	6.66 × 10^−160^
Ndufa9	PAS_chr3_1188	8199532	Hypothetical protein	−1.0381	5.51 × 10^−229^	--	--
Ndufs2	PAS_chr3_0808	8200498	Hypothetical protein	−1.0048	1.02 × 10^−157^	--	--
Ndufa6	PAS_chr4_0535	8201285	Hypothetical protein	−1.0031	6.90 × 10^−124^	--	--
SDHD	PAS_chr3_0424	8199556	Membrane anchor subunit of succinate dehydrogenase	−1.3199	3.86 × 10^−139^	−1.4013	1.19 × 10^−148^
ISP	PAS_chr2-1_0850	8198393	Hypothetical protein	−1.2036	0	--	--
Cyt1	PAS_chr3_0997	8199742	Cytochrome c1	−1.2324	3.89 × 10^−174^	−1.1014	8.88 × 10^−171^
QCR2	PAS_chr2-2_0430	8199133	Subunit 2 of the ubiquinol Cytochrome-c reductase complex	−1.1952	2.78 × 10^−246^	−1.0056	3.22 × 10^−204^
QCR7	PAS_chr1-1_0322	8196587	Subunit 7 of the ubiquinol Cytochrome-c reductase complex	−1.199	3.64 × 10^−298^	−1.0103	7.86 × 10^−205^
QCR8	PAS_chr1-4_0313	8197763	Subunit 8 of ubiquinol Cytochrome-c reductase complex	−1.0855	1.41 × 10^−265^	--	--
QCR9	PAS_chr4_0520	8201463	Hypothetical protein	−1.125	2.36 × 10^−58^	--	--
QCR10	PAS_chr2-1_0717	8198335	Hypothetical protein	−1.4053	6.25 × 10^−212^	−1.3829	1.80 × 10^−136^
COX10	PAS_chr1-3_0194	8196833	Heme A: farnesyltransferase	−1.2751	7.51 × 10^−119^	−1.0158	3.44 × 10^−78^
COX7A	PAS_chr2-2_0265	8198670	Hypothetical protein	−1.5686	5.23 × 10^−35^	−1.7976	6.61 × 10^−42^
COX7C	PAS_chr2-2_0266	8198671	Subunit VIII of cytochrome c oxidase	−1.1987	3.73 × 10^−43^	−1.0889	3.07 × 10^−25^
COX11	PAS_chr2-1_0226	8198685	Mitochondrial inner membrane protein required for delivery of copper to the Cox1p subunit of cytochrome C	−1.4389	4.15 × 10^−163^	−1.3231	5.35 × 10^−138^
COX7A	PAS_chr2-1_0746	8198951	Hypothetical protein	−1.378	3.59 × 10^−114^	−1.4629	1.67 × 10^−104^
COX5B	PAS_chr2-1_0361	8199049	Subunit IV of cytochrome c oxidase	−1.2454	1.34 × 10^−99^	−1.415	2.08 × 10^−124^
COX4	PAS_chr3_0615	8199817	Subunit Va of cytochrome c oxidase	−1.2259	0	−1.2926	0
COX5A	PAS_chr3_0824	8200317	Subunit VI of cytochrome c oxidase	−1.4422	0	−1.6052	0
COX6B	PAS_chr4_0422	8200705	Subunit VIb of cytochrome c oxidase	−1.9104	0	−2.2113	0
COX15	PAS_chr4_0449	8201398	Protein required for the hydroxylation of heme O to form heme A	−1.4388	1.40 × 10^−318^	−2.1416	0
COX6A	PAS_chr2-1_0363	8198106	Subunit VIa of cytochrome c oxidase	--	--	−1.0228	4.76 × 10^−53^
epsilon	PAS_chr2-1_0612	8198937	Hypothetical protein	−1.2169	2.50 × 10^−212^	−1.1389	7.07 × 10^−138^
k	PAS_chr3_0161	8199436	Hypothetical protein	−1.3219	1.50 × 10^−50^	−1.3717	3.29 × 10^−37^
β	PAS_chr2-2_0165	8198741	β subunit of the F1 sector of mitochondrial F1F0 ATP synthase	−1.2684	1.44 × 10^−170^	−1.2277	3.37 × 10^−182^
α	PAS_chr3_0576	8199781	α subunit of the F1 sector of mitochondrial F1F0 ATP synthase	−1.029	9.73 × 10^−114^	--	-
g	PAS_chr3_0819	8200313	Subunit g of the mitochondrial F1F0 ATP synthase	−1.1936	1.58 × 10^−125^	−1.1555	1.52 × 10^−124^
Ppase /EC 3.6.1.1	PAS_chr1-3_0070	8197380	Mitochondrial inorganic pyrophosphatase	−1.2811	5.87 × 10^−273^	−1.2728	7.19 × 10^−257^

A dash line indicates no significant change in comparison to BI.

**Table 3:** List of DEGs encoding proteins of peroxisome in PI1 and PI2 in comparison to BI.

Protein Name	Gene Name	Gene ID	Gene Description	Log_2_ (Fold Change)/PI1 vs. BI	*p*_adj_/PI1 vs. BI	Log_2_ (Fold Change)/PI2 vs. BI	*p*_adj_/PI2 vs. BI
PEX1	PAS_chr3_1045	8200007	Peroxisome biosynthesis protein PAS1	2.6156	0	2.7808	0
PEX2	PAS_chr3_0043	8199329	Peroxisomal integral membrane protein	2.3827	0	2.8031	0
PEX3	PAS_chr3_1073	8200033	Peroxisomal membrane protein (PMP)	1.4397	3.017 × 10^−310^	1.92	0
PEX5	PAS_chr2-2_0186	8198761	Peroxisomal membrane signal receptor for the C-terminal tripeptide signal sequence (PTS1)	2.949	0	3.5365	0
PEX6	PAS_chr1-4_0133	8197117	AAA-peroxin that heterodimerizes with AAA-peroxin Pex1p	2.0075	0	2.1995	0
PEX13	PAS_chr2-2_0207	8198615	Integral peroxisomal membrane protein required for the translocation of peroxisomal matrix proteins	3.7578	0	4.7576	0
PEX14	PAS_chr4_0794	8200572	Peroxisomal membrane peroxin that is a central component of the peroxisomal protein import machinery	2.2643	0	2.8084	0
PMP34	PAS_chr3_0099	8199380	Mitochondrial NAD+ transporter, involved in the transport of NAD^+^ into the mitochondria	4.9561	0	5.2536	0
PXMP4	PAS_chr1-1_0352	8196615	Hypothetical protein	1.1228	3.11 × 10^−18^	1.2063	1.85 × 10^−49^
ACOX	PAS_chr1-4_0538	8197432	Fatty-acyl coenzyme A oxidase	2.3146	0	2.5487	0
ACSL	PAS_chr4_0352	8200793	Long chain fatty acyl-CoA synthetase	2.9342	0	3.3382	0
ACSL	PAS_chr2-1_0785	8198062	Long chain fatty acyl-CoA synthetase	1.092	0	1.0212	4.13 × 10^−262^
CRAT	PAS_chr1-4_0074	8197291	Outer mitochondrial carnitine acetyltransferase, minor ethanol-inducible enzyme	−1.0294	1.17 × 10^−225^	−1.1661	8.13 × 10^−269^
CAT	PAS_chr2-2_0131	8198267	Catalase A	2.1646	0	2.509	0
SOD	PAS_chr4_0786	8200564	Cytosolic superoxide dismutase	1.8615	0	2.353	0
SOD	PAS_chr4_0788	8200566	Mitochondrial ribosomal protein of the small subunit	1.0854	1.35 × 10^−45^	--	--
MVK	PAS_chr1-3_0187	8197654	Mevalonate kinase	−1.0019	6.82 × 10^−64^	−1.1063	7.71 × 10^−74^
IDH	PAS_chr1-1_0233	8196735	Mitochondrial NADP-specific isocitrate dehydrogenase	−1.0698	4.44 × 10^−202^	−1.0284	4.63 × 10^−179^
IDH	PAS_chr2-1_0580	8198933	Cytosolic NADP-specific isocitrate dehydrogenase	−2.9882	4.16 × 10^−277^	−3.1716	2.87 × 10^−311^
XDH	PAS_chr2-2_0112	8199216	Hypothetical protein - xanthine dehydrogenase	1.3445	0	1.36	0
ABCD	PAS_chr2-2_0272	8198676	Subunit of a heterodimeric peroxisomal ATP-binding cassette transporter complex	--	--	1.227	1.19 × 10^−160^
PEX12	PAS_chr4_0759	8200652	C3HC4-type RING-finger peroxisomal membrane peroxin	--	--	1.7009	0

A dash line indicates no significant change in comparison to BI.

**Table 4:** List of DEGs related with protein production and degradation in PI1 and PI2 in comparison to BI.

Protein Name	Gene Name	Gene ID	Gene Description	Log_2_ (Fold Change)/PI1 vs. BI	*p*_adj_/PI1 vs. BI	Log_2_ (Fold Change)/PI2 vs. BI	*p*_adj_/PI2 vs. BI
MAT1	PAS_chr4_0204	8201110	Subunit of TFIIH and nucleotide excision repair factor 3 complexes	3.3693	0	4.0208	0
TFIIH1	PAS_chr4_0745	8200638	Subunit of TFIIH and nucleotide excision repair factor 3 complexes	−1.6733	6.87 × 10^−286^	−2.1074	0
ARF-BP1	PAS_chr3_0044	8200180	E3 ubiquitin ligase of the hect-domain class	1.479	2.52 × 10^−178^	1.94	0
UBE2G2	PAS_chr3_0924	8200276	Ubiquitin conjugating enzyme, involved in the ER-associated protein degradation pathway	−1.6354	5.35 × 10^−176^	−1.7258	1.56 × 10^−195^
Apc3	PAS_chr1-3_0148	8197615	Subunit of the Anaphase-Promoting Complex/Cyclosome (APC/C)	1.1055	2.11 × 10^−39^	1.2557	1.02 × 10^−59^
HIP2	PAS_chr4_0429	8200711	Ubiquitin-conjugating enzyme that mediates selective degradation of short-lived and abnormal protein	−1.0662	1.72 × 10^−135^	−1.035	1.75 × 10^−123^
Ubc6/7	PAS_chr3_0924	8200276	Ubiquitin conjugating enzyme, involved in the ER-associated protein degradation pathway	−1.6354	5.35 × 10^−176^	−1.7258	1.56 × 10^−195^
Ubc7	PAS_chr3_0924	8200276	Ubiquitin conjugating enzyme, involved in the ER-associated protein degradation pathway	−1.6354	5.35 × 10^−176^	−1.7258	1.56 × 10^−195^
Ubx2	PAS_chr1-1_0084	8197210	Protein involved in ER-associated protein degradation	−1.1051	3.95 × 10^−82^	−1.146	5.05 × 10^−84^
PDIs	PAS_chr1-1_0160	8196664	Protein disulfide isomerase, multifunctional protein resident in the endoplasmic reticulum lumen	−1.3541	2.3 × 10^−295^	−1.5559	0
Hsp40	PAS_chr2-2_0066	8199171	Protein chaperone involved in regulation of the HSP90 and HSP70 functions	1.3014	1.248 × 10^−319^	1.1231	3.2 × 10^−221^
Sec20	PAS_chr1-4_0462	8197050	Membrane glycoprotein v-SNARE	−1.0588	1.18 × 10^−57^	--	--
Stx1-4	PAS_chr1-4_0294	8197744	Plasma membrane t-SNARE involved in fusion of secretory vesicles at the plasma membrane	−1.1446	2.29 × 10^−200^	−1.3775	3.9 × 10^−264^
Cul4	PAS_chr1-4_0609	8197739	Cullin, structural protein of SCF complexes	--	--	1.0004	3.17 × 10^−46^

A dash line indicates no significant change in comparison to BI.

## Supplementary Materials

The following are available online at https://www.mdpi.com/2073-4425/10/8/606/s1, Figure S1: Reproducibility and reliability analyses of each sample (BI, PI1, PI2). The log_10_(FPKM + 1) were applied to calculate the Pearson correlation coefficient R^2^. The sequencing data with a coefficient R^2^ > 0.92 was regarded as high quality, Figure S2: Overview of DEGs for each comparison groups. (**A**), comparison group: PI1 versus BI; (**B**), comparison group: PI2 versus BI; (**C**), comparison group: PI2 versus PI1. The horizontal axis displays the fold change of expression levels of DEGs in different groups, and the vertical axis shows the statistical significance of this variation. Red dots represent genes which are up-regulated, and green dots means the down-regulated parts, Figure S3: Cluster analysis of DEGs in different comparison groups. Sample names are marked at the bottom. Clustering with log_10_ (FPKM + 1); red, high-expression level genes; blue, low-expression level genes, Figure S4: Functional annotations of DEGs between different sample groups. (**A**) Comparable group: PI1 and BI; (**B**) Comparable group: PI2 and BI; (**C**) Comparable group: PI2 and PI1. The green bars represent biological process; orange bars represent cellular component; purple bars represent molecular function, Figure S5: Description of DEGs involved in the amino acid biosynthesis process in PI1 and PI2 in comparison to BI. Up-regulated or down-regulated genes in comparison to BI (*p*_adj_ < 0.01) are marked with *up* or *down arrow* (black arrow stands for sample PI1, red arrow indicates PI2), respectively. A *dash* line indicates no significant change in comparison to BI, Table S1: Overall quality of the high-throughput sequencing data for each sample, Table S2: Proportion of clean reads mapping to the reference genome, Table S3: Proportion of detected genes at different expression levels, Table S4: List of DEGs involved in amino acid biosynthesis in PI1 and PI2 in comparison to BI, Table S5: Information of DEGs related to the central carbon metabolism, amino acid biosynthesis and function of peroxisome in PI2 in comparison to PI1.
