# Supplementary material for: Transcriptomic Analysis of the Influence of Methanol Assimilation on the Gene Expression in the Recombinant Pichia pastoris Producing Hirudin Variant 3
Source: Genes (Basel). 2019 Aug 12;10(8):606. doi: 10.3390/genes10080606 (PMC6722669; doi:10.3390/genes10080606)

Journal name:

Genes

Manuscript title:

Transcriptomic Analysis of the Influence of Methanol Assimilation on the Gene Expression in the Recombinant *Pichia pastoris* Producing Hirudin Variant 3

Author list:

Tao Li [1, 2], Jieying Ma [1], Zehua Xu [1], Shuang Wang [1], Nan Wang [1], Shulin Shao [1], Wei Yang [2], Lin Huan [1] and Yihan Liu[1,*]

The affiliations and addresses of the authors:

[1]Key Laboratory of Industrial Fermentation Microbiology, Ministry of Education, Tianjin Key Laboratory of Industrial Microbiology, The College of Biotechnology, Tianjin University of Science and Technology, Tianjin 300457, China

[2]College of Basic Science, Tianjin Agricultural University, Tianjin 300384, China

Address: No.22, Jinjing Road, Xiqing District, Tianjin, 300384, China.

* Corresponding author address: No. 29, 13th Avenue, Tianjin Economic and Technological Development Area, Tianjin 300457, China.

The e-mail address, telephone and fax numbers of the corresponding author:

E-mail: lyh@tust.edu.cn (Yihan Liu)

Tel:+86 22 60601958; fax: +86 22 60602298.

Table S1 Overall quality of the high-throughput sequencing data for each sample

Sample name	Raw reads	Clean reads	clean bases	Error rate (%)	Q20(%)	Q30(%)
BI_1	48423150	46883296	7.03G	0.02	96.82	92.11
BI_2	47420250	45920584	6.89G	0.02	96.85	92.14
BI_3	41617514	39290632	5.89G	0.02	95.98	90.64
PI1_1	49227724	46403092	6.96G	0.02	96.2	90.94
PI1_2	44371436	41808438	6.27G	0.02	95.91	90.38
PI1_3	46491252	43878414	6.58G	0.02	96.13	90.82
PI2_1	47175090	44444988	6.67G	0.02	95.94	90.43
PI2_2	41181634	39720186	5.96G	0.02	95.46	88.92
PI2_3	42263776	40630120	6.09G	0.02	95.64	89.53

[a] Q20, Q30 refer to the proportion of base calls with Phred scores >20 or 30 in the total bases.

Table S2 Proportion of clean reads mapping to the reference genome

Sample name	BI_1	BI_2	BI_3	PI1_1	PI1_2	PI1_3	PI2_1	PI2_2	PI2_3
Total reads	46883296	45920584	39290632	46403092	41808438	43878414	44444988	39720186	40630120
Total mapped	44327073 (94.55%)	43381041 (94.47%)	36718356 (93.45%)	43088741 (92.86%)	38872086 (92.98%)	40698966 (92.75%)	41246265 (92.8%)	36763909 (92.56%)	37564783 (92.46%)
Multiple mapped	58339 (0.12%)	52106 (0.11%)	39051 (0.1%)	85722 (0.18%)	66755 (0.16%)	72729 (0.17%)	88184 (0.2%)	66639 (0.17%)	70181 (0.17%)
Uniquely mapped	44268734 (94.42%)	43328935 (94.36%)	36679305 (93.35%)	43003019 (92.67%)	38805331 (92.82%)	40626237 (92.59%)	41158081 (92.6%)	36697270 (92.39%)	37494602 (92.28%)
Reads map to '+'	22111597 (47.16%)	21641075 (47.13%)	18318580 (46.62%)	21484079 (46.3%)	19386970 (46.37%)	20296324 (46.26%)	20563073 (46.27%)	18324619 (46.13%)	18723904 (46.08%)
Reads map to '-'	22157137 (47.26%)	21687860 (47.23%)	18360725 (46.73%)	21518940 (46.37%)	19418361 (46.45%)	20329913 (46.33%)	20595008 (46.34%)	18372651 (46.26%)	18770698 (46.2%)
Non-splice reads	43436428 (92.65%)	42541098 (92.64%)	35999883 (91.62%)	42342684 (91.25%)	38203728 (91.38%)	39966086 (91.08%)	40510529 (91.15%)	36128313 (90.96%)	36873126 (90.75%)
Splice reads	832306 (1.78%)	787837 (1.72%)	679422 (1.73%)	660335 (1.42%)	601603 (1.44%)	660151 (1.5%)	647552 (1.46%)	568957 (1.43%)	621476 (1.53%)

Table S3 Proportion of detected genes at different expression levels

FPKM Interval	BI_1	BI_2	BI_3	PI1_1	PI1_2	PI1_3	PI2_1	PI2_2	PI2_3
0～1	14(0.28%)	11(0.22%)	12(0.24%)	11(0.22%)	12(0.24%)	11(0.22%)	11(0.22%)	11(0.22%)	12(0.24%)
1～3	44(0.87%)	44(0.87%)	44(0.87%)	14(0.28%)	16(0.32%)	16(0.32%)	17(0.34%)	16(0.32%)	14(0.28%)
3～15	781(15.49%)	751(14.89%)	748(14.84%)	435(8.63%)	437(8.67%)	422 (8.37%)	400(7.93%)	398(7.89%)	408(8.09%)
15～60	2015(39.96%)	2023(40.12%)	2011(39.88%)	2285(45.32%)	2297(45.56%)	2294(45.50%)	2253(44.68%)	2248(44.59%)	2248(44.59%)
>60	2188(43.40%)	2213(43.89%)	2227(44.17%)	2297(45.56%)	2280(45.22%)	2299(45.60%)	2361(46.83%)	2369(46.99%)	2360(46.81%)

Table S4 List of DEGs involved in amino acid biosynthesis in PI1 and PI2 in comparison to BI

EC No.	Gene name	Gene ID	Gene description	Log_2(fold change)/ PI1 vs BI	p_{adj}/ PI1 vs BI	Log_2(fold change)/ PI2 vs BI	p_{adj} / PI2 vs BI
1.5.1.7	PAS_chr1-1_0447	8196649	Saccharopine dehydrogenase	-1.4546	0	-1.2705	0
1.1.1.42	PAS_chr1-1_0233	8196735	Isocitrate dehydrogenase	-1.0698	4.44×10^{-202}	-1.0284	4.63×10^{-179}
2.7.1.11	PAS_chr1-4_0047	8196884	Phosphofructokinase	-1.4239	0	-1.8729	0
2.5.1.54	PAS_chr1-4_0218	8196893	3-deoxy-D-arabino-heptulosonate-7-p hosphate synthase	-2.0437	0	-2.6741	0
2.2.1.1	PAS_chr1-4_0150	8197134	Transketolase	-1.3666	5.53×10^{-118}	-1.3961	2.01×10^{-134}
3.6.1.31/ 3.5.4.19/ 1.1.1.23	PAS_chr1-4_0160	8197144	Multifunctional enzyme HIS4	-1.739	0	-2.7775	0
4.1.2.13	PAS_chr1-1_0072	8197200	Fructose1,6-bisphosphate aldolase	-1.9342	3.70×10^{-282}	-2.0982	0
2.3.3.1	PAS_chr1-1_0475	8197246	Hypothetical protein	-1.3184	1.47×10^{-121}	-1.3199	4.41×10^{-131}
2.6.1.39	PAS_chr1-4_0608	8197738	Aromatic aminotransferase I	-1.7326	0	-1.8652	0
2.7.2.3	PAS_chr1-4_0292	8197742	3-phosphoglycerate kinase	-2.1073	0	-2.8743	0
2.7.1.40	PAS_chr2-1_0769	8198046	Pyruvate kinase	-1.3692	0	-1.9631	0
2.7.2.8	PAS_chr2-1_0168	8198090	Protein that is processed in the mitochondrion to yield acetylglutamate kinase and	-1.1606	8.28×10^{-237}	-1.3693	1.28×10^{-303}

			N-acetyl-gamma-gl				
1.1.1.87	PAS_chr2-2_0168	8198271	Homo-isocitrate dehydrogenase	-2.5135	0	-2.5685	0
2.5.1.54	PAS_chr2-1_0473	8198396	3-deoxy-D-arabino-heptulosonate-7-phosphate synthase	-1.3479	5.81×10^{-173}	-1.1056	7.83×10^{-119}
2.1.1.14	PAS_chr2-1_0160	8198543	Cobalamin-independent methionine synthase	-2.2734	0	-2.5412	0
4.2.1.22	PAS_chr2-2_0137	8198715	Cystathionine beta-synthase	-1.4955	0	-2.4844	0
2.7.1.11	PAS_chr2-1_0402	8198870	Phosphofructokinase	-1.759	0	-2.711	0
2.3.3.13	PAS_chr2-1_0415	8198883	Alpha-isopropylmalate synthase	-1.166	2.26×10^{-286}	-1.728	0
1.2.1.12	PAS_chr2-1_0437	8198905	Glyceraldehyde-3-phosphate dehydrogenase	-1.9318	1.00×10^{-134}	-2.3868	4.91×10^{-185}
1.1.1.42	PAS_chr2-1_0580	8198933	Cytosolic NADP-specific isocitrate dehydrogenase	-2.9882	4.16×10^{-277}	-3.1716	2.8774×10^{-311}
4.4.1.8	PAS_chr2-1_0358	8199046	Cystathionine beta-lyase	-1.4861	1.14×10^{-93}	-1.2	2.05×10^{-72}
4.2.3.5	PAS_chr2-1_0637	8199251	Bifunctional chorismate synthase and flavin reductase	-1.2805	2.11×10^{-95}	-1.6786	3.74×10^{-138}
1.1.1.95	PAS_chr2-1_0657	8199271	3-phosphoglycerate dehydrogenase	-1.7569	0	-2.1751	0
1.1.1.85	PAS_chr3_0039	8199325	Beta-isopropylmalate dehydrogenase	-2.5699	0	-2.6204	0
4.2.1.11	PAS_chr3_0082	8199366	Enolase I	-1.9763	7.89×10^{-307}	-2.5303	0

2.3.1.35	PAS_chr3_0176	8199450	Ornithine acetyltransferase	-1.5173	$1.87×10^{−291}$	-1.6039	$7.26×10^{−307}$
2.6.1.2	PAS_chr3_0482	8199609	Putative alanine transaminase	-2.059	0	-2.4058	0
4.1.1.48	PAS_chr3_0962	8199709	Exhibiting indole-3-glycerol-phosphate synthase and anthranilate synthase activity	-1.336	$5.30×10^{−247}$	-1.6567	$7.99×10^{−316}$
2.1.3.3	PAS_chr3_0623	8199823	Ornithine carbamoyltransferase	-2.0192	$1.76×10^{−141}$	-2.2192	$2.94×10^{−156}$
4.2.1.20	PAS_chr3_0634	8199834	Tryptophan synthase	-1.0847	$4.62×10^{−187}$	-1.1716	$1.58×10^{−204}$
4.2.1.20	PAS_chr2-1_0679	8199292	Tryptophan synthase	--	--	-1.1046	$1.64×10^{−172}$
2.5.1.9	PAS_chr3_0506	8199874	Pentafunctional arom protein	-1.253	$2.51×10^{−136}$	-1.5084	$8.18×10^{−182}$
1.5.1.10	PAS_chr3_0528	8199896	Saccharopine dehydrogenase	-1.8479	0	-2.073	0
2.6.1.52	PAS_chr3_0566	8199930	3-phosphoserine aminotransferase	-1.5013	$3.40×10^{−318}$	-1.6803	0
2.5.1.54	PAS_chr3_0936	8200288	3-deoxy-D-arabino-heptulosonate-7-p hosphate	-1.5277	0	-2.2264	0
5.4.2.1	PAS_chr3_0826	8200319	Tetrameric phosphoglycerate mutase	-1.8358	0	-2.0814	0
5.4.2.1	PAS_chr3_0693	8200393	Tetrameric phosphoglycerate mutase	-1.0681	$4.40×10^{−55}$	-1.2688	$2.30×10^{−74}$
4.2.1.36	PAS_chr4_0795	8200573	Homoaconitase	-1.2235	$1.37×10^{−204}$	-1.4603	$5.37×10^{−271}$
2.1.2.1	PAS_chr4_0415	8200698	Cytosolic serine hydroxymethyltransferase	-1.3783	$1.01×10^{−232}$	-1.276	$2.32×10^{−223}$
2.5.1.47	PAS_chr4_0330	8200771	Methionine and cysteine synthase	-2.3956	$1.53×10^{−232}$	-2.3737	$1.08×10^{−242}$

2.3.1.1	PAS_chr1-4_0215	8196890	Acetylglutamate synthase	1.092	1.07×10^{-62}	1.1929	6.93×10^{-53}
2.7.2.11	PAS_chr3_0294	8200122	Gamma-glutamyl kinase	1.3619	4.16×10^{-92}	1.0425	1.14×10^{-53}
5.3.1.6	PAS_chr4_0212	8200883	Ribose-5-phosphate ketol-isomerase	6.5546	0	6.9045	0
5.3.1.6	PAS_chr4_0213	8200884	Ribose-5-phosphate ketol-isomerase	1.8505	0	2.1047	4.19×10^{-314}
5.4.99.5	PAS_chr4_0050	8201141	Chorismate mutase	3.1313	0	3.2774	0
3.5.3.1	PAS_chr4_0684	8201391	Arginase responsible for arginine degradation	1.0927	5.61×10^{-190}	1.3903	1.81×10^{-304}
3.5.3.1	PAS_chr2-2_0288	8198188	Arginase responsible for arginine degradation	--	--	1.3401	1.65×10^{-174}
4.4.1.1	PAS_chr1-4_0489	8197076	Cystathionine gamma-lyase	--	--	-1.3342	1.19×10^{-237}
2.6.1.1	PAS_chr4_0974	8201006	Hypothetical protein	--	--	1.2759	0
2.6.1.11	PAS_chr4_0645	8201353	Acetylornithine aminotransferase	--	--	-1.2908	2.34×10^{-256}

A *dash* line indicates no significant change in comparison to BI.

Table S5 Information of DEGs related to the central carbon metabolism, amino acid biosynthesis and function of peroxisome in PI2 in comparison to PI1

Protein name/EC No.	Gene name	Gene ID	Gene description	Log_2(fold change)/ PI2 vs PI1	p_{adj}/ PI2 vs PI1
			Carbon metabolism		
2.7.1.1	PAS_chr4_0624	8201333	Non-essential protein of unknown function required for transcriptional induction	-1.0488	0
2.7.1.1	PAS_chr1-4_0561	8197692	Hexokinase-2	-1.1398	0
			Oxidative phosphorylation		
--	--	--	--	--	--
			Amino acid biosynthesis		
2.6.1.11	PAS_chr4_0645	8201353	Acetylornithine aminotransferase	-1.0169	1.64×10^{-130}
3.6.1.31/ 3.5.4.19/ 1.1.1.23	PAS_chr1-4_0160	8197144	Multifunctional enzyme HIS4	-1.037	0
			Protein production and degradation related genes		
--	--	--	--	--	--
			Peroxisome		
PEX13	PAS_chr2-2_0207	8198615	Integral peroxisomal membrane required for the translocation of	1.0013	4.47×10^{-275}

peroxisomal matrix proteins

A *dash* line indicates no significant change in comparison to BI.

Figure S1 Reproducibility and reliability analyses of each sample (BI, PI1, PI2). The log_{10}(FPKM+1) were applied to calculate the Pearson correlation coefficient R^2. The sequencing data with a coefficient R^2 >0.92 was regarded as high quality.

Figure S1

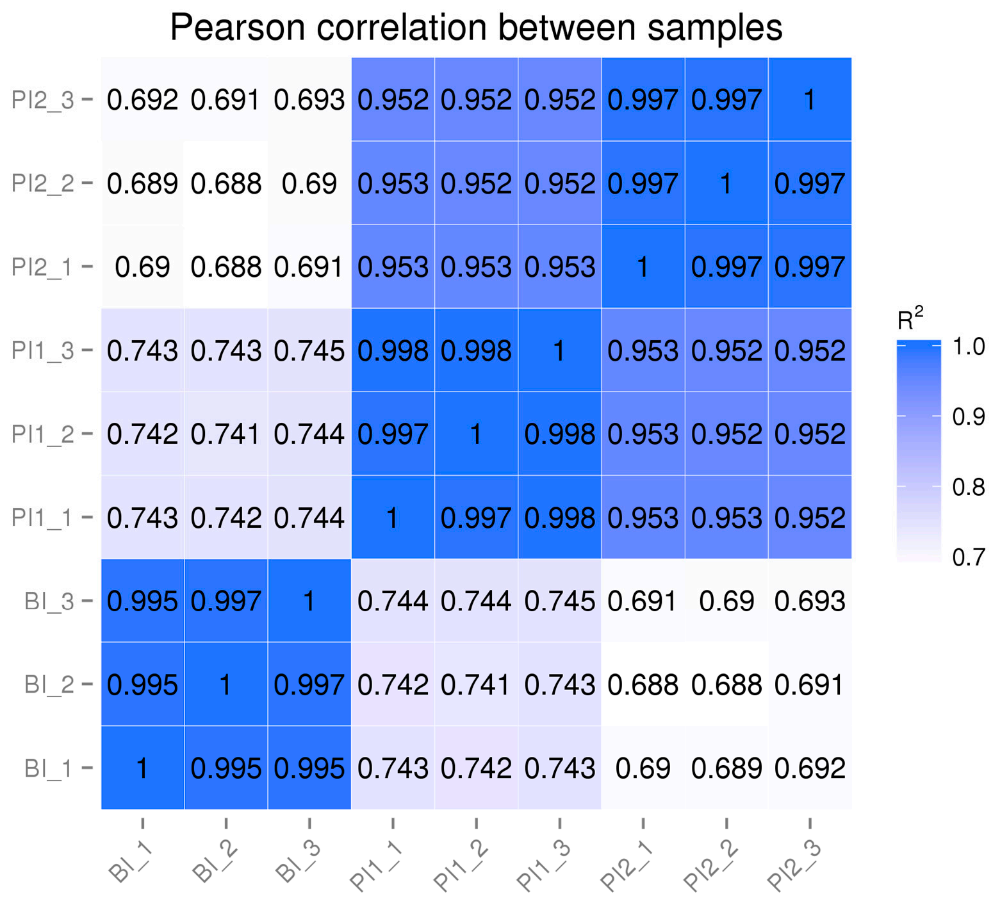

Figure S2 Overview of DEGs for each comparison groups. **A**, comparison group: PI1 versus BI; **B**, comparison group: PI2 versus BI; **C**, comparison group: PI2 versus PI1. The horizontal axis displays the fold change of expression levels of DEGs in different groups, and the vertical axis shows the statistical significance of this variation. Red dots represent genes which are up-regulated, and green dots means the down-regulated parts.

Figure S2

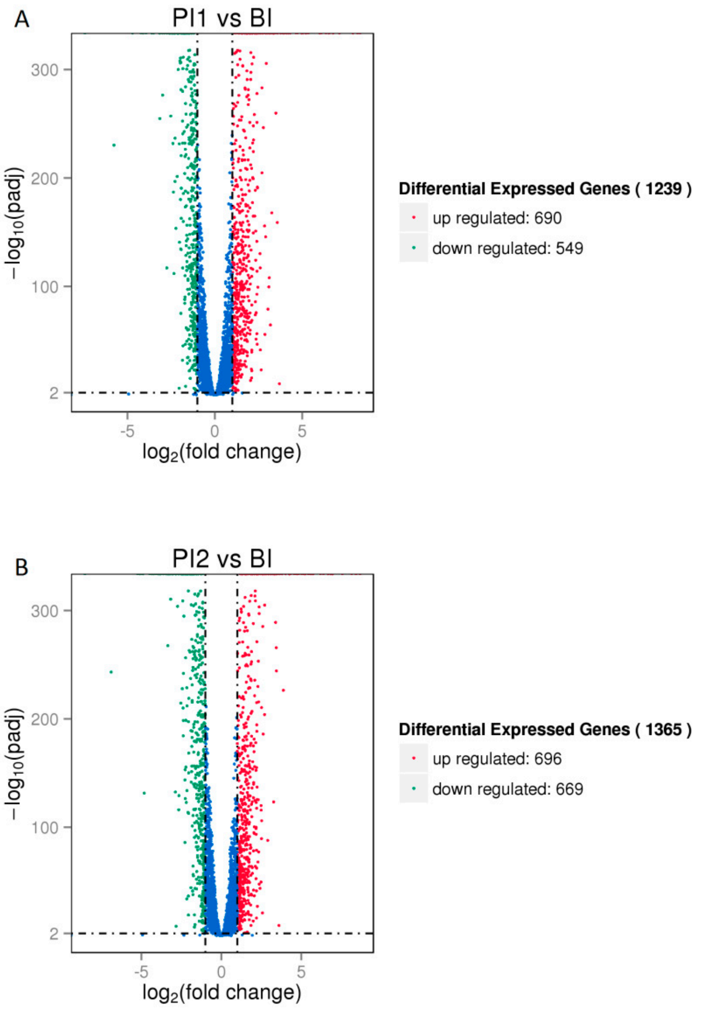

C

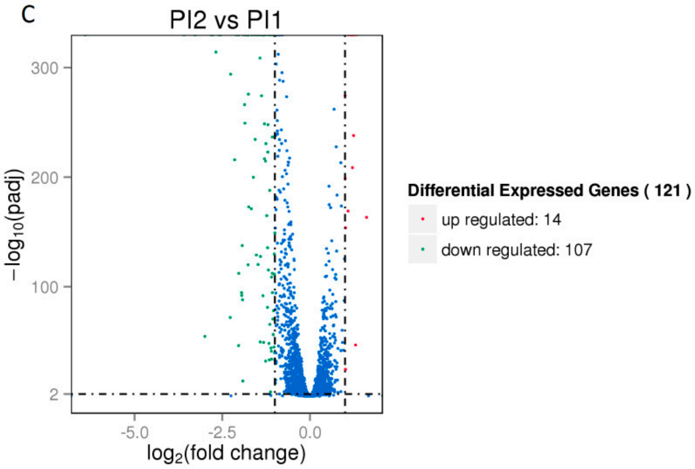
PI2 vs PI1
300
200
100
2
−log10(padj)
-5.0
-2.5
0.0
log2(fold change)
Differential Expressed Genes (121)
up regulated: 14
down regulated: 107

Figure S3 Cluster analysis of DEGs in different comparison groups. Sample names are marked at the bottom. Clustering with $\log_{10}$ (FPKM + 1); red, high-expression level genes; blue, low-expression level genes.

Figure S3

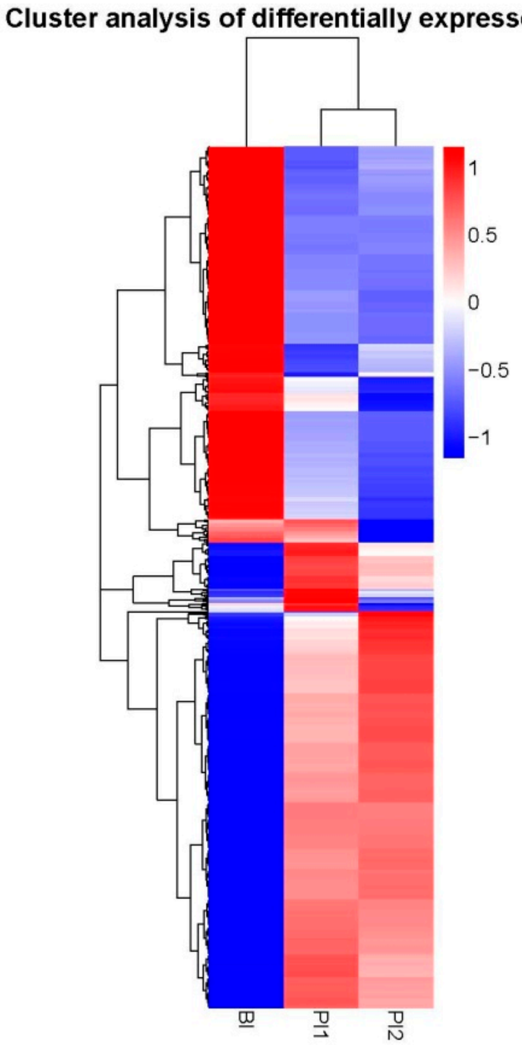

Figure S4 Functional annotations of DEGs between different sample groups. **A** Comparable group: PI1 and BI; **B** Comparable group: PI2 and BI; **C** Comparable group: PI2 and PI1. The green bars represent biological process; orange bars represent cellular component; purple bars represent molecular function.

Figure S4

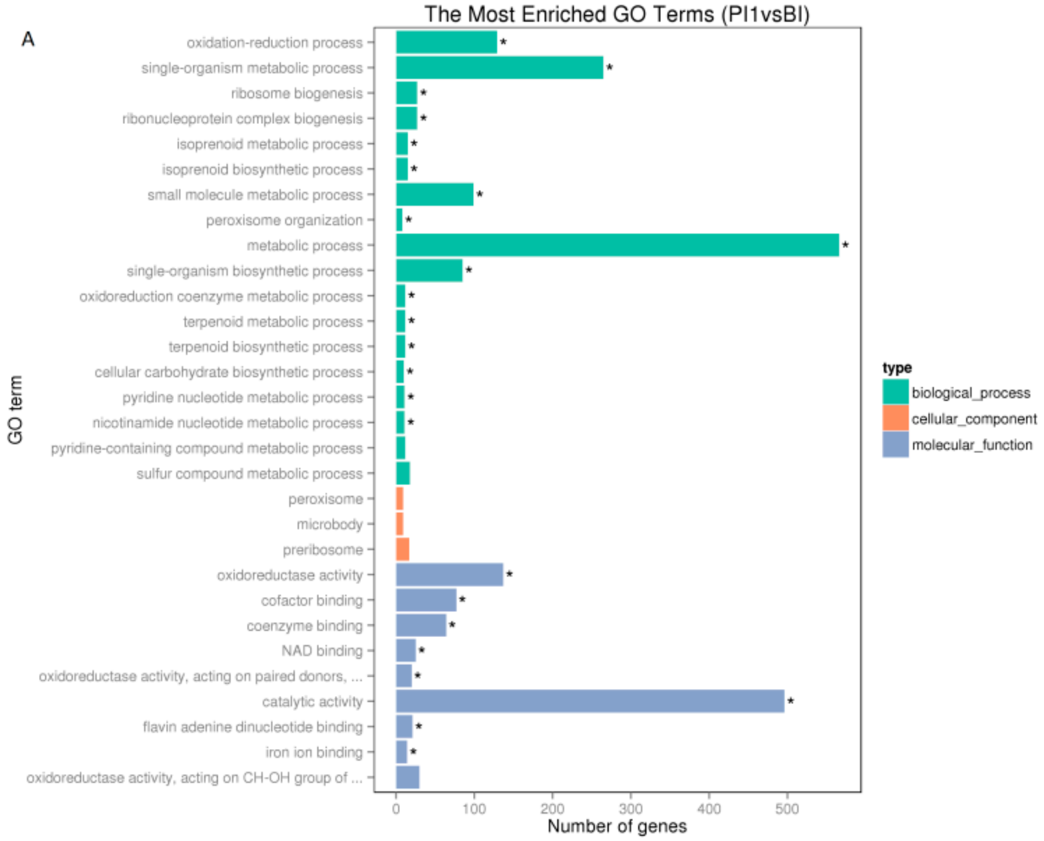

B

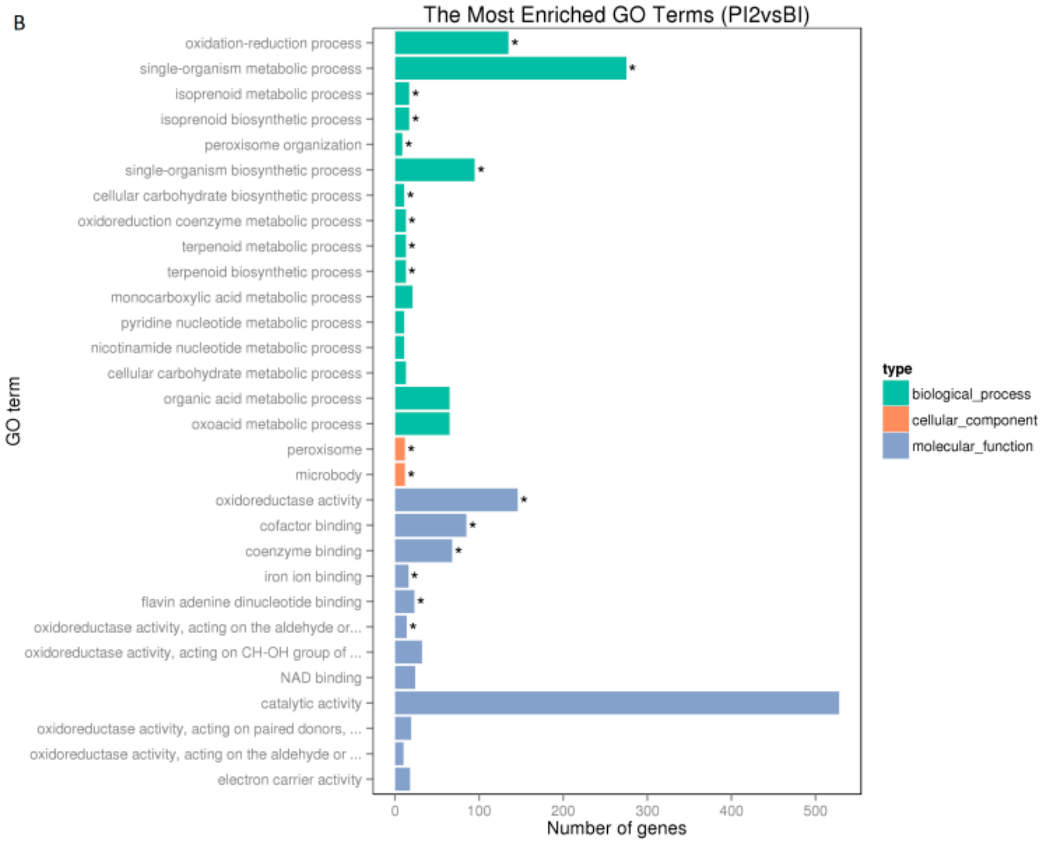

The Most Enriched GO Terms (PI2vsBI)
oxidation-reduction process
single-organism metabolic process
isoprenoid metabolic process
isoprenoid biosynthetic process
peroxisome organization
single-organism biosynthetic process
cellular carbohydrate biosynthetic process
oxidoreduction coenzyme metabolic process
terpenoid metabolic process
terpenoid biosynthetic process
monocarboxylic acid metabolic process
pyridine nucleotide metabolic process
nicotinamide nucleotide metabolic process
cellular carbohydrate metabolic process
organic acid metabolic process
oxoacid metabolic process
peroxisome
microbody
oxidoreductase activity
cofactor binding
coenzyme binding
iron ion binding
flavin adenine dinucleotide binding
oxidoreductase activity, acting on the aldehyde or...
oxidoreductase activity, acting on CH-OH group of ...
NAD binding
catalytic activity
oxidoreductase activity, acting on paired donors, ...
oxidoreductase activity, acting on the aldehyde or ...
electron carrier activity
GO term
0
100
200
300
400
500
Number of genes
type
biological_process
cellular_component
molecular_function

C

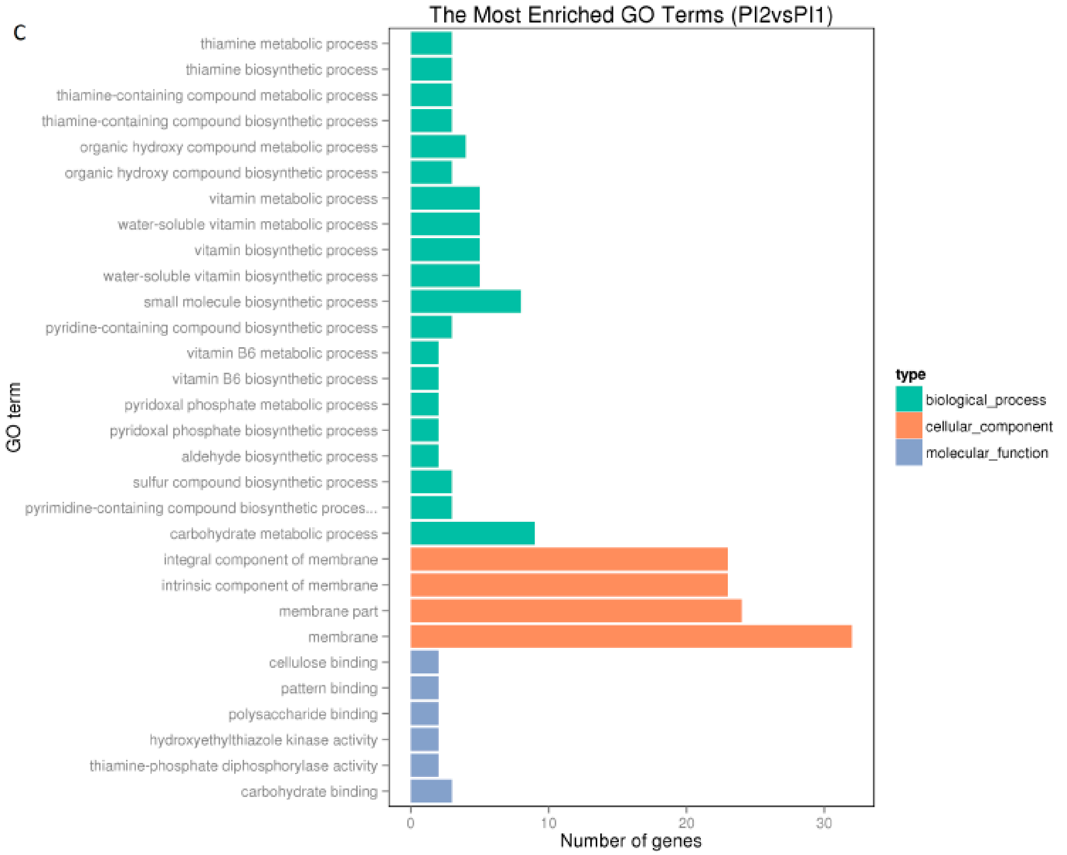

The Most Enriched GO Terms (PI2vsPI1)
thiamine metabolic process
thiamine biosynthetic process
thiamine-containing compound metabolic process
thiamine-containing compound biosynthetic process
organic hydroxy compound metabolic process
organic hydroxy compound biosynthetic process
vitamin metabolic process
water-soluble vitamin metabolic process
vitamin biosynthetic process
water-soluble vitamin biosynthetic process
small molecule biosynthetic process
pyridine-containing compound biosynthetic process
vitamin B6 metabolic process
vitamin B6 biosynthetic process
pyridoxal phosphate metabolic process
pyridoxal phosphate biosynthetic process
aldehyde biosynthetic process
sulfur compound biosynthetic process
pyrimidine-containing compound biosynthetic proces...
carbohydrate metabolic process
integral component of membrane
intrinsic component of membrane
membrane part
membrane
cellulose binding
pattern binding
polysaccharide binding
hydroxyethylthiazole kinase activity
thiamine-phosphate diphosphorylase activity
carbohydrate binding
GO term
0
10
20
30
Number of genes
type
biological_process
cellular_component
molecular_function

Figure S5 Description of DEGs involved in the amino acid biosynthesis process in PI1 and PI2 in comparison to BI. Up-regulated or down-regulated genes compared with BI (p_{adj} < 0.01) are marked with *up* or *down arrow* (black arrow stands for sample PI1, red arrow indicates PI2), respectively. A *dash* line indicates no significant change in comparison to BI.

Figure S5

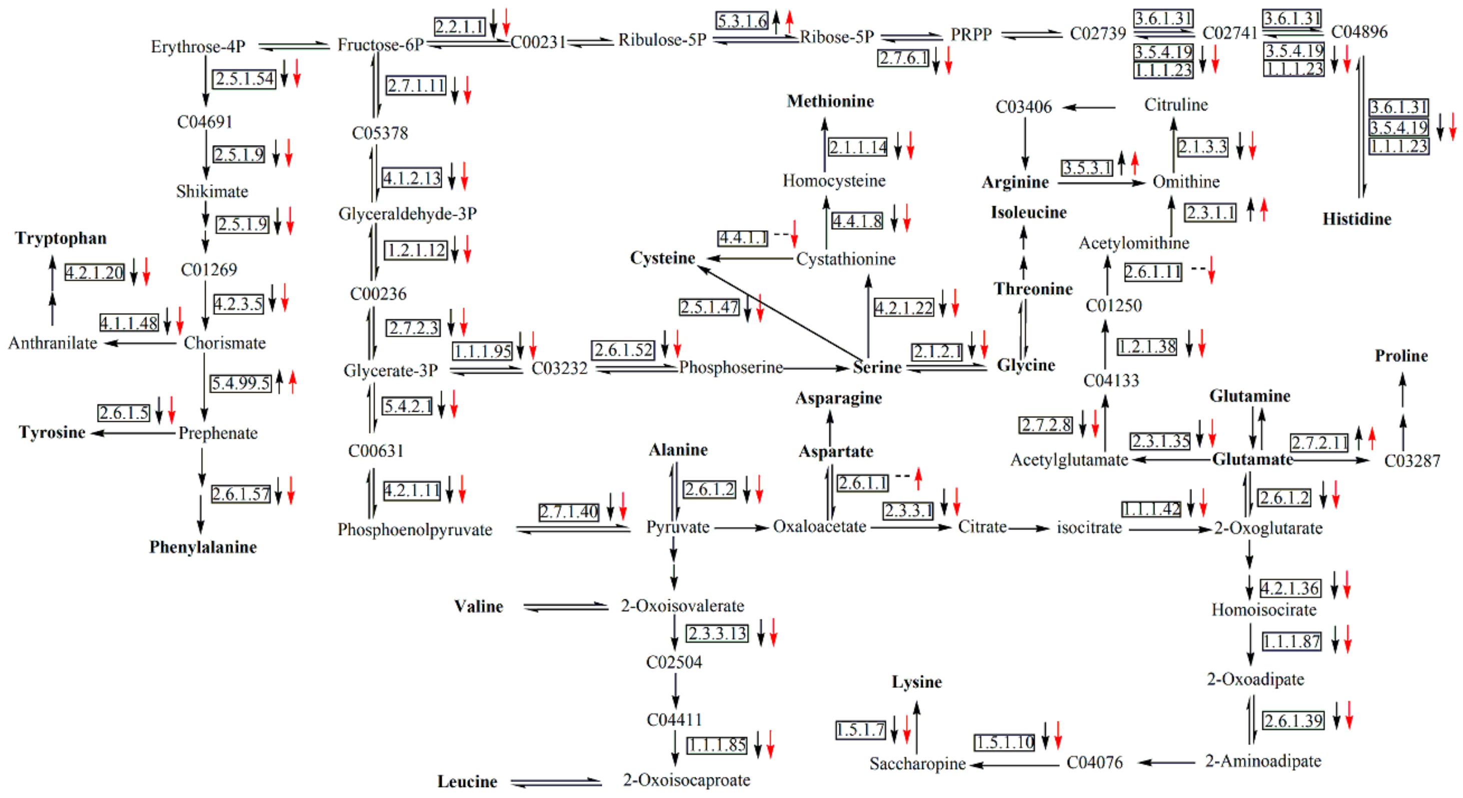

Supplement: Supplementary file 1 [file genes-10-00606-s001.pdf]
